# Evaluation of Green Strategies for Prolonging the Lifespan of Linear Wireless Sensor Networks

**DOI:** 10.3390/s24217024

**Published:** 2024-10-31

**Authors:** Valery Nkemeni, Fabien Mieyeville, Godlove Suila Kuaban, Piotr Czekalski, Krzysztof Tokarz, Wirnkar Basil Nsanyuy, Eric Michel Deussom Djomadji, Musong L. Katche, Pierre Tsafack, Bartłomiej Zieliński

**Affiliations:** 1 Faculty of Engineering and Technology, University of Buea, Buea P.O. Box 63, Cameroon; 2 University de Lyon, Université Claude Bernard Lyon 1, Ecole Centrale de Lyon, INSA Lyon, CNRS, Ampère, F-69621 Villeurbanne, France; fabien.mieyeville@univ-lyon1.fr; 3 Institute of Theoretical and Applied Informatics, Polish Academy of Sciences, Baltycka 5, 44-100 Gliwice, Poland; gskuaban@iitis.pl; 4 Faculty of Automatic Control, Electronics and Computer Science, Silesian University of Technology, Akademicka 16, 44-100 Gliwice, Poland; piotr.czekalski@polsl.pl (P.C.); krzysztof.tokarz@polsl.pl (K.T.);; 5 College of Technology, University of Buea, Buea P.O. Box 63, Cameroon

**Keywords:** energy management, energy conservation, energy balancing, energy harvesting, linear wireless sensor networks, green wireless sensor networks, network lifetime

## Abstract

Battery-powered sensor nodes encounter substantial energy constraints, especially in linear wireless sensor network (LWSN) applications like border surveillance and road, bridge, railway, powerline, and pipeline monitoring, where inaccessible locations exacerbate battery replacement challenges. Addressing these issues is crucial for extending a network’s lifetime and reducing operational costs. This paper presents a comprehensive analysis of the factors affecting WSN energy consumption at the node and network levels, alongside effective energy management strategies for prolonging the WSN’s lifetime. By categorizing existing strategies into node energy reduction, network energy balancing, and energy replenishment, this study assesses their effectiveness when implemented in LWSN applications, providing valuable insights to assist engineers during the design of green and energy-efficient LWSN monitoring systems.

## 1. Introduction

### 1.1. Wireless Sensor Networks and Applications

A wireless sensor network (WSN) comprises numerous embedded nodes equipped with sensing, processing, and wireless communications functionalities strategically deployed across a designated area to observe physical or environmental conditions [1]. As distributed systems, WSNs utilize wireless communication for inter-node communication, rendering them suitable for a wide range of applications.

**Figure 1:**
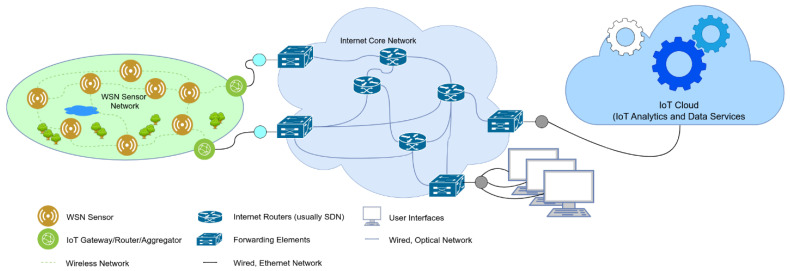
Relationship between IoT and WSN.

In tandem with WSNs, the concept of the Internet of Things (IoT) has emerged. The IoT can be defined as the interconnection of identifiable devices within an internet network for the purpose of sensing and monitoring processes [2]. WSN is thus a central component of the IoT [3], as the IoT leverages the capabilities of WSNs to collect data about the environment and execute actions based on the analysis of the gathered data [4]. In contrast to WSNs, which do not inherently require internet connectivity, the IoT predominantly utilizes internet protocol (IP) connectivity to assign each of its components, or “things”, a unique address. Figure 1 depicts the relationship between the IoT and WSN, illustrating a typical IoT scenario where data collection is facilitated using WSNs.

**Figure 2:**
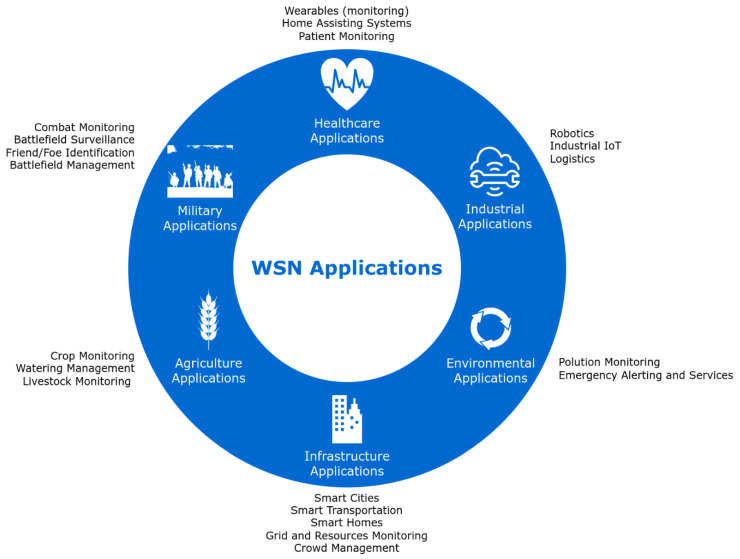
Application areas for WSN.

The application areas of WSNs encompass a wide range of domains, including geographical monitoring, habitat monitoring, transportation, military systems, business processes, structural health monitoring, microclimate research, medical care, and others [5,6]. In a survey conducted by Kandris et al. [7], the applications of WSNs were classified into six primary categories (military, environmental, health, flora and fauna, industrial, and urban) based on their respective usage scenarios, as illustrated in Figure 2.

### 1.2. Linear Wireless Sensor Networks

**Figure 3:**
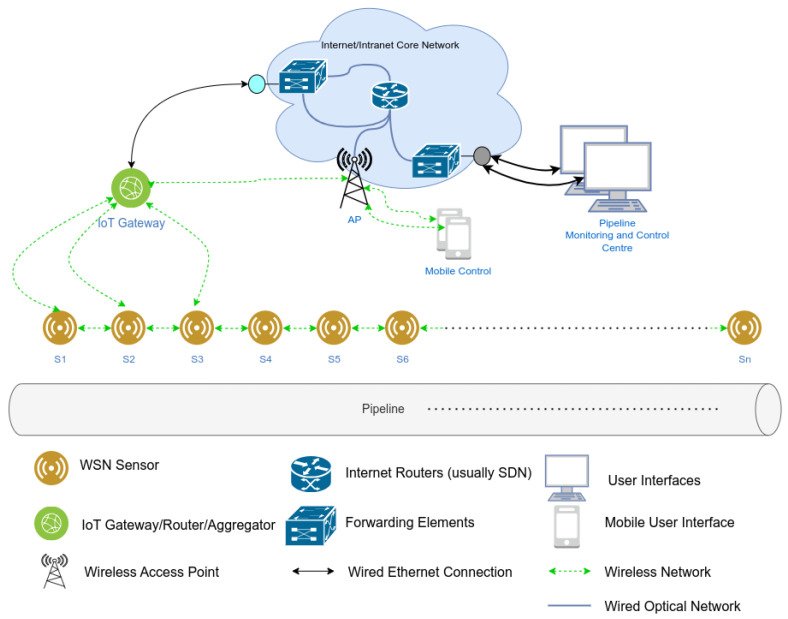
LWSN deployed for pipeline monitoring.

In many WSN applications, the structure of the monitored area necessitates a linear deployment of sensor nodes, giving rise to a special class of WSNs known as linear wireless sensor networks (LWSNs) [8]. Prominent examples include border surveillance [9,10], road monitoring [11], railway/subway monitoring [12,13], powerline monitoring [14,15,16], sea/river shore monitoring [17], and pipeline monitoring [18,19,20,21,22,23,24]. In all the aforementioned applications, the common characteristic is that the area under consideration extends solely in one dimension [25]. Figure 3 depicts a LWSN deployed for pipeline monitoring.

Linear wireless sensor networks (LWSNs) present several challenges, such as ensuring successful end-to-end delivery, providing a reasonable packet delivery timeframe, and maintaining energy efficiency. The main reason for this is that the linear topology restricts the number of neighbors and, consequently, the potential transmission routes, making data delivery more vulnerable to failure compared to traditional WSNs. In addition to critical node failures (caused by energy exhaustion of nodes closer to the sink due to an uneven load distribution), failures can also arise from an increased number of retransmissions, leading to higher packet collision rates and traffic congestion [26,27]. Furthermore, LWSNs usually span over long distances [28], and the deployment of nodes in LWSNs often occurs in remote or inaccessible areas, such as mountain ranges, overhead transmission lines, under water pipelines, etc. [29], posing difficulties for battery replacement when the nodes become depleted [30]. Replacing the batteries of sensor nodes deployed in such areas presents significant challenges, including being labor-intensive and logistically complex to access the sensor nodes [31]. Moreover, the associated costs of frequent battery replacements contribute to the project’s operational expenses, making energy efficiency a prominent concern in such applications. Additionally, depleted batteries directly hinder the project’s goal of achieving continuous monitoring over extended periods, which may compromise data collection and analysis efforts. Addressing this challenge has given rise to an active research area referred to as green WSN/IoT [32,33,34,35]. In green WSNs, alternative power sources (energy harvesting) and energy-efficient (energy savings) strategies are used to minimize the reliance on battery power and prolong the operational lifespan of sensor nodes. This reduces the frequency of battery replacement, reduce the amount of electronic waste (pollution), and also reduce the carbon footprint. Therefore, prioritizing energy efficiency is crucial in the design of LWSN solutions and by effectively addressing energy consumption challenges, LWSN solutions can extend their operational lifetimes and enhance their reliability in monitoring and data collection applications.

### 1.3. Contributions of This Paper

Numerous studies in the literature have reviewed energy management strategies for extending the lifespan of WSNs [34,36,37,38]. However, due to the unique topology of LWSNs, the challenges and solutions pertinent to LWSNs differ from those of conventional WSNs [25]. Consequently, strategies effective for conventional WSNs may not be directly applicable to LWSNs. Hence, there is a need to explore energy management strategies specifically tailored to prolong the lifespan of LWSNs. To the best of our knowledge, no existing study has comprehensively evaluated energy management strategies for enhancing the lifespan of LWSNs. This paper aims to address this gap by first providing a comprehensive review of energy management strategies used in traditional WSNs and then evaluating their suitability and impact in LWSNs. It highlights the ineffectiveness of certain energy management techniques designed for traditional WSNs when used in the context of LWSNs.

The contributions of this paper are threefold:

1. Novel Classification and Evaluation of Energy Management Techniques for LWSNs: This paper provides a comprehensive taxonomy of energy management techniques specifically tailored to LWSNs. Unlike traditional WSNs, LWSNs possess unique structural and operational challenges, particularly in critical applications such as border surveillance, pipeline monitoring, powerline monitoring, etc. This research is among the first to categorize and evaluate energy conservation strategies, balancing methods, and energy replenishment solutions within this context, providing a roadmap for engineers and researchers working on green LWSN deployments.
2. Significance in Prolonging the LWSN’s Lifespan: Extending the operational lifespan of battery-powered LWSNs is crucial due to the high cost and logistical challenges associated with battery replacement in remote or inaccessible areas. This study goes beyond reviewing energy management strategies; it also offers insights into the effectiveness of each technique under the constraints of LWSNs. By identifying the techniques that significantly reduce energy consumption and by evaluating their feasibility in LWSNs, this study contributes essential knowledge that will enhance the reliability and sustainability of such networks.
3. Practical Implications for a Green LWSN Design: The findings of this research contribute directly to the design of eco-friendly LWSNs by minimizing reliance on battery replacements and reducing the environmental impact. This study identifies specific energy management techniques that are both feasible and effective in LWSNs. The proposed taxonomy and analysis offer practical guidelines for network designers to develop energy-efficient solutions in environmentally sensitive and economically challenging regions.

### 1.4. Organization of This Paper

The remainder of the paper is organized as follows: Section 2 presents a framework for classifying LWSNs, and Section 3 presents a classification of the energy management techniques aimed at extending a WSN’s lifetime. Section 4, Section 5 and Section 6 delve into energy conservation techniques, energy-balancing techniques, and energy-harvesting techniques, respectively, evaluating the effectiveness of the techniques in the context of LWSNs. Section 7 discusses the challenges associated with adopting energy management techniques from conventional WSNs in LWSNs. Finally, Section 8 concludes the paper.

## 2. Framework of Linear Wireless Sensor Networks

### 2.1. Classification of LWSNs

A LWSN is a special class of WSN, characterized by its distinct topology, where nodes are arranged in a straight or near-straight line, creating unique challenges in routing, energy management, and network reliability [8]. Unlike traditional WSNs, which are often designed for multi-dimensional topologies or random deployments, LWSNs are optimized for applications where monitoring follows a linear path, making energy efficiency and routing optimization critical [39]. In addition to its distinct topology, LWSNs also differ from traditional WSNs in terms of node heterogeneity. While traditional WSNs may feature homogeneous nodes with similar roles, LWSNs often comprise a mix of different types of nodes.

Nodes in a LWSN are categorized based on their roles in the network, which impacts how data are gathered and transmitted. Jawhar et al. [8] identified three primary types of nodes in LWSNs:

1. Basic Sensor Nodes (BSNs): These are the standard sensing units in the network. Their primary function is to collect data from the environment and transmit it to other nodes for further processing. BSNs are typically energy-constrained, with limited communication ranges and computing power, making energy efficiency essential for extending the network’s lifespan.
2. Data Relay Nodes (DRNs): These nodes act as intermediaries between the BSNs and the data dissemination nodes. Their function is to forward data collected by BSNs along the linear path, ensuring that data reach their final destination (the central processing system). DRNs help maintain network connectivity over long distances, and they typically have more power than BSNs.
3. Data Dissemination Nodes (DDNs): These are high-level nodes responsible for collecting data from DRNs and transmitting the data to the base station or central monitoring system. They have stronger processing capabilities and higher energy reserves than the other types of nodes, enabling them to manage large amounts of data and handle long-range communication. These nodes make use of long-range wireless communication technologies such as LoRa, satellite, cellular, etc.

According to the study by Jawhar et al. [8], LWSNs can be classified both topologically and hierarchically. A LWSN from a given topological category can belong to any of the three hierarchical categories depending on the application.

From a topological point-of-view, the authors classified LWSNs under three categories: thin, thick, and very thick LWSNs. In thin LWSNs, sensor nodes are sparsely deployed along the linear path, resulting in minimal coverage and reduced redundancy. These networks are suitable for applications where only a basic level of monitoring is required, such as pipeline and powerline monitoring [40]. Thick LWSNs feature a moderate density of nodes along the monitored path, providing greater redundancy and more robust monitoring. They are commonly used in scenarios requiring higher data reliability, such as railway or highway monitoring [39]. Very thick LWSNs have a high density of sensor nodes, ensuring comprehensive coverage and redundancy. These networks are typically used in critical infrastructure monitoring, such as border surveillance or high-security installations, where data loss and network failure are unacceptable [41].

From a hierarchical point-of-view, the authors classified LWSNs under three categories: one-level, two-level, and three-level. In one-level LWSNs, there is no hierarchy among the nodes, with all nodes having the same role (sensing, aggregation, and compression) and transmitting data directly to a central base station or processing node. Two-level LWSNs introduce a basic hierarchical structure, where BSNs relay data to DRNs, which perform aggregation and then forward the information to a DDN for further processing. In three-level LWSNs, a more complex hierarchy is established, with multiple layers of data transmission. BSNs relay data to DRNs, which aggregate the data and send it to DDNs for transmission to the central system. This hierarchical design improves network efficiency and reduces the load on individual sensor nodes.

### 2.2. The Need for Specialized Protocols in LWSNs

The presentation of LWSNs provided in Section 2.1 shows that the most significant difference between traditional WSNs and LWSNs lies in the network topology and routing. Traditional WSNs are typically deployed in multi-dimensional or randomly distributed configurations, allowing nodes to have multiple neighboring nodes and offering more flexibility in routing and data transmission. LWSNs, on the other hand, are constrained by their linear deployment, where each node has only a few immediate neighbors along the path.

From a routing point-of-view, the overheads required in traditional WSNs for tasks such as route discovery, route maintenance, handling node failures to ensure network reliability, and managing node heterogeneity are significantly reduced in LWSNs due to their well-defined and structured topology [8]. In traditional WSNs, nodes are often deployed randomly across a wide area, requiring complex routing protocols to establish multi-path and multi-hop communications, deal with dynamic network changes, and ensure robust data delivery. In contrast, LWSNs feature a linear topology where nodes are typically placed along a defined path (e.g., pipelines, borders, or roads), meaning the routing paths are more straightforward and predictable. This defined structure simplifies routing, as nodes generally forward data in a unidirectional or bidirectional manner along the line. Consequently, the need for frequent route updates and resource-intensive discovery processes is minimized, leading to lower overheads [42]. Additionally, the concept of shortest path routing, which is commonly used in traditional WSNs, is not well-suited for LWSNs because of the linear configuration that limits the routing options to either forwarding data to the left or to the right. Given this constrained topology, routing decisions are less about finding the shortest path and more about ensuring reliable data transmission along the linear structure while minimizing energy consumption [28]. Lastly, fault tolerance mechanisms can be more straightforwardly implemented, e.g., using the opposite direction of the failed node to reach the sink or increasing the transmission power to jump over the failed sensor node and reach the next node along the line [8]. The focus is on maintaining linear connectivity, reducing the need for energy-intensive recovery protocols, and optimizing energy consumption in LWSNs [43]. Therefore, LWSNs require specialized routing protocols that can account for the limited routing possibilities, focusing on factors such as node energy levels, link quality, and fault tolerance to extend the network’s operational lifetime [30].

### 2.3. Hypothesis on Energy Management Techniques in LWSNs

Energy management techniques commonly employed in traditional WSNs, particularly those targeting the optimization of network topology and routing layers, may not be as effective in LWSNs due to the constrained and unique linear topology. We hypothesize that the energy management techniques that involve the network layer will be most impacted by the differences between traditional WSNs and LWSNs. Consequently, only WSN energy management techniques that directly affect the network layer are likely to have a considerable impact when applied to LWSNs, requiring a rethinking of strategies to accommodate the network’s linear structure. Also, the most impactful energy management techniques in LWSNs will be those that are capable of reducing the number of multi-hop communications in LWSNs.

In this study, we focus only on thin LWSNs. Instead of focusing on a hierarchical network structure, such as those found in clustered networks, our interest lies in flat LWSNs, where all sensor-generated messages are relayed in a multi-hop fashion toward the sink node. The following sections present a comprehensive review of the green strategies and energy management techniques used in traditional WSNs and evaluate their applicability to LWSNs. By categorizing these techniques into node energy reduction, network energy balancing, and energy harvesting, we analyze the effectiveness of every technique in each of the categories when applied to the specific requirements of LWSNs.

## 3. Classification of Energy Management Techniques for Prolonging WSN’s Lifetime

In this section, we begin by examining the energy consumption patterns within a WSN, followed by an exploration of the various taxonomies used to classify the energy management techniques in WSNs from previous studies. We conclude the section by introducing a taxonomy that we have adopted for categorizing energy management techniques aimed at prolonging the lifespan of WSNs.

### 3.1. Energy Consumption Analysis of WSN

To comprehensively study the range of energy management techniques applicable to WSNs, it is important to first analyze the power dissipation characteristics of a sensor node and identify the factors that influence energy consumption both at the node level and across the entire network. Conducting a thorough and systematic analysis of energy consumption within a sensor node is crucial for identifying the key parameters that impact WSN energy consumption. This analysis aids in gaining a deeper understanding of how various energy management strategies function to effectively minimize energy consumption and extend the lifespan of WSNs. In this subsection, we undertake an examination of WSN energy consumption by first modeling the energy consumption of a sensor node. Subsequently, we outline the parameters influencing energy consumption within a WSN, both at the node level and across the network as a whole, as well as the sources of energy wastage.

#### 3.1.1. Analysis of the Energy Consumption of a Sensor Node

**Figure 4:**
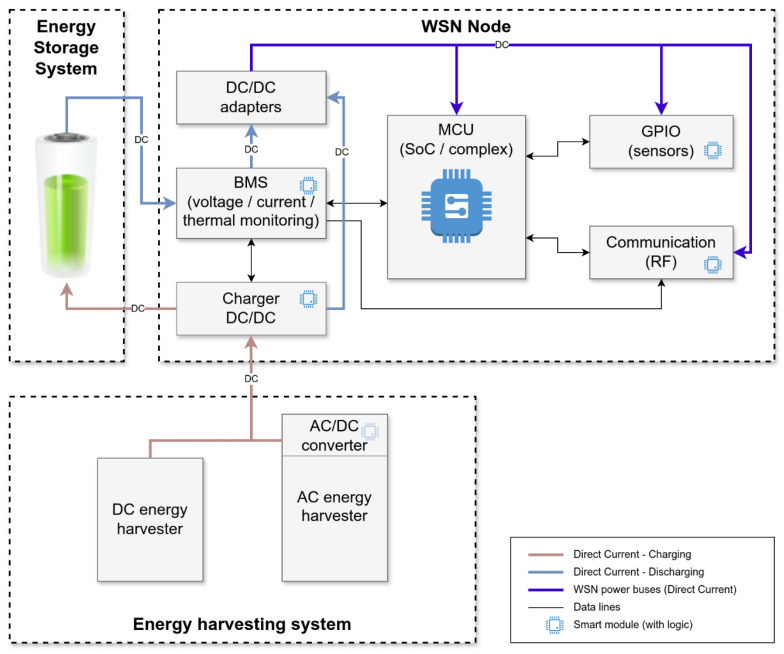
Components of a sensor node.

A wireless sensor node comprises a sensing unit, processing unit, communication unit, and power supply unit, as depicted in Figure 4.

- The sensing unit serves as an interface between the real world and the digital world. It detects different phenomena from the environment, ranging from light, heat, pressure, acceleration, etc., which serve as inputs to the sensor nodes. It is usually made up of transducer and signal conditioning (ADC, filter, and amplifier) parts. The transducer generates an electric signal proportional to the event or condition being monitored or measured [44], and the generated electric signal is typically converted to digital form using the ADC since the processing unit of sensor nodes can only process digital data [45].
- The processing unit is the core of a wireless sensor node, and it is involved with the collection of data from the sensors, processing this data (data filtering, data compression, data aggregation, data routing, etc.), deciding when and where to send it, reception of data from other sensor nodes, and the setting of actuators’ behaviors (if they are present). It has to execute various programs, ranging from time-critical signal processing to communication protocols of application programs. The categories of processing and control units used in a sensor node include the microcontroller (MCU), digital signal processor (DSP), programmable gate arrays (FPGA), and application-specific integrated circuits (ASIC) [32,44].
- The communication unit is in charge of sending and receiving packets to or from other sensor nodes in the network via wireless communication. The transceiver has different modes of operation, which include transmitting, receiving, and idle/sleep modes, with each state consuming a different amount of energy [46]. The choice of the communication unit is very crucial in determining the sensor node’s energy consumption since this unit consumes the highest energy compared to the processing and sensing units [1,47].
- The power supply unit provides the node with the energy required to cater to the node’s operations (sensing, data processing, and communication). Sensor nodes are usually powered via energy stored in batteries or capacitors in applications that require deployment in areas without access to the power grid. The batteries used can either be rechargeable or non-rechargeable. For long-lasting WSN applications where there is a need for sensor nodes to go for long periods unattended and without replacing their energy source, the limited energy storage capacity of batteries is not attractive. Currently, most sensor nodes are designed to have the optional ability to recharge their battery from energy harvested through scavenging techniques such as photovoltaics, temperature gradients, vibrations, pressure variations, the flow of air/liquid, etc. [48,49].

To model the energy consumption of a sensor node, it is essential to account for the energy consumed by its various components. The energy consumed by a sensor node is the sum of the energy consumed in the active and inactive (sleep) states.

(1) $$E_{total}=\underset{k}{\sum }\left(E_{Active,k}+E_{Sleep,\;k}\right)$$

where $E_{total}\;$ is the total energy, $E_{Active,k}$ and $\;E_{Sleep,\;k}\;$ represent the energy consumed in the active and sleep states in a single time step (cycle) *k*, respectively.

Assuming that at every cycle (time step *k*), which lasts for a total duration of *T*, the sensor node undergoes a wake-up from sleep for a duration of $T_{WU,\;k}$, performs measurements for a duration of $T_{SU,\;k}$, performs data processing for a duration of $T_{proc,\;k}$, takes $T_{WUT,\;k}$ to wake the transceiver up from sleep, perform data transmission and data reception for $T_{TX,\;k}$ and $T_{RX,\;k}$, respectively, and sleeps for a duration of $T_{Sleep,\;k}$ [50,51], then

(2) $$T=T_{WU,k}+T_{SU,\;k}+T_{proc,\;k}+T_{WUT,\;k}+T_{TX,\;k}+T_{RX,\;k}+T_{Sleep,\;k}$$

The energy consumed in the sleep state is given by

(3) $$E_{Sleep,k}=P_{Sleep,k}\times T_{Sleep,\;k}$$

where $P_{Sleep,k}\;\mathrm{and}\;T_{Sleep,\;k}\;$ are the power consumption and the time duration in the sleep mode, respectively, at time step *k*.

The energy consumed in the active state is the sum of the energy consumed by the constituent parts of the sensor node and is given by

(4) $$E_{Active,k}=E_{PU,k}+E_{CU,\;k}+E_{SU,\;k}$$

where $E_{PU,k},\;E_{CU,\;k},\;\mathrm{and}\;E_{SU,\;k}$ are the energy consumed by the sensor node’s processing unit, communication unit, and sensing unit, respectively, at time step *k*.

The processor’s energy consumption, $E_{PU,k}$, is derived by adding the switching (dynamic) and leakage (static) energies in the circuits. Dynamic energy is the energy needed to activate parasitic capacitors on an IC from a digital zero voltage to a digital one voltage, while static energy is the energy dissipated as a result of the current leakage from power to ground that occurs constantly in the circuitry [52]. The energy consumed by the processing unit is given by

(5) $$E_{PU,k}=P_{PU,k}\times T_{PU,\;k}+E_{st,k}$$

where $P_{PU,k}$ is the power consumed by the processing unit when it is active. This power is dependent on the operational frequency ($f_{PU,\;k}$) of the processing unit. $E_{st,k}$ is the leakage (static) energy, and $T_{PU,\;k}$ is the total duration for which the processing unit is active.

(6) $$T_{PU,k}=T_{WU,k}+T_{SU,\;k}+T_{proc,\;k}+T_{WUT,\;k}+T_{TX,\;k}+T_{RX,\;k}$$

(7) $$P_{PU,k}=\;C\times V^{2}\times f_{PU,\;k}$$

where C and V denote the switching capacitance and supply voltage, respectively, and $f_{PU,\;k}$ is the operational frequency of the processing unit.

A major part of the processing unit’s energy is consumed during data processing. The duration for data processing ($T_{proc,\;k}$) is dependent on the operational frequency ($f_{PU,\;k}$) of the processing unit and the number of instructions ($N_{inst}$). Thus, the energy consumption of the processor unit in the active state depends on the number of processed bits and the frequency of the processor based on the following equation:

(8) $$T_{proc,k}=\frac{N_{inst}}{f_{PU,k}}$$

Since the processing unit encompasses the memory unit in our model, the energy consumption of the processing unit is affected by the number of stored bits, the number of memory reads and writes, and the duration of storage.

The energy consumed by the sensing unit is given by

(9) $$E_{SU,k}=P_{SU,k}\times T_{SU,k}$$

where $P_{SU,k}$ and $T_{SU,k}$ represent the power of the sensing device and the duration of sensing, respectively. The energy consumption of the sensing unit is dependent on the sensor’s coverage radius, the data generation rate, and the number of generated bits.

The energy consumed by the communication unit is given by

(10) $$E_{CU,k}=E_{TX,k}+E_{RX,k}$$

where $E_{TX,k}$ and $E_{RX,k}$ are the power of the transceiver when operating in the transmit and receive modes, respectively.

The sensor node’s transmission energy model is given from [52] as:

(11) $$E_{TX,k}=P_{WUT,k}\times T_{WUT,k}+\frac{L}{R}\left[P_{TX}+\left(\frac{P_{RX}\times A\times d^{n}}{\eta }\right)\right]$$

where $P_{WUT,k}$ and $T_{WUT,k}$ represent the starting power and starting time of the transceiver, $P_{TX}$ is the power of the transceiver in the transmitting mode, *L* is the length of the packet transmitted/received, *R* is the data rate, $P_{RX}\times A\times d^{n}$ is the power sent to the antenna of the transmitting node, in which $P_{RX}$ is the power received by the antenna of the receiving node and delivered to the low noise amplifier (LNA), *A* is determined by characteristics of the transmitting and receiving antennas, *n* is the path loss exponent, which depends on the nature of the clutter type, and η is the drain efficiency of the power amplifier (PA).

The sensor node’s reception energy model is given from [52] as:

(12) $$E_{RX,k}=P_{WUT,k}\times T_{WUT,k}+\frac{L}{R}P_{RX}+L\times E_{dec}$$

where $P_{RX,k}$ is the power of the transceiver in the reception mode, and $E_{dec}$ is the energy consumed in decoding a single bit.

Combing Equations (11) and (12), the energy consumed by the communication is given by

(13) $$E_{CU,k}=P_{WUT,k}\times T_{WUT,k}+\frac{L}{R}\left[P_{TX}+\left(\frac{P_{RX}\times A\times d^{n}}{\eta }\right)\right]+\frac{L}{R}P_{RX}+L\times E_{dec}$$

The energy consumption of the communication unit for digital signal processing in an active state depends on the number of received and transmitted bits and the amount of energy needed for coding and decoding packets [53].

This energy consumption analysis is focused at the node level, making the modeling applicable to both traditional WSNs and LWSNs.

After modeling the energy consumption of a wireless sensor node, the following subsection will present the parameters that influence the energy consumption of a WSN and also the sources of energy wastage in the WSN.

#### 3.1.2. Parameters Influencing Energy Consumption of WSN

There are several parameters that affect the energy consumption of a WSN. These parameters can have an influence on the energy consumption of the WSN, either at the node level or at the network-wide level [54]. In this subsection, we present the parameters that affect the energy consumption of a WSN at both the node and network-wide levels and the sources of energy waste in a WSN.

The parameters that affect the energy consumption of the WSN at the sensor node level are parameters that have a direct influence on the energy consumption of the sensor node. These parameters belong to the physical layer and basically operate on individual nodes. They encompass both hardware and software parameters [55]. Understanding and optimizing these parameters is essential for designing energy-efficient WSNs that can prolong the network’s lifespan and enhance its overall performance [56]. Table 1 presents a list of parameters and the components of the sensor node that is affected.

The parameters listed in Table 1 are focused at the node level and thus have a similar effect on both traditional WSNs and LWSNs. However, we recognize that the parameters influencing energy consumption at the network-wide level pertain to the entire network rather than individual nodes. Optimizing these parameters at the network-wide level is crucial for enhancing energy efficiency and extending the lifespan of WSNs while also meeting the application requirements and ensuring reliable data delivery. However, their optimization may be less effective in LWSNs. Table 2 outlines the parameters that influence energy consumption at the network-wide level, identifies the corresponding network layers involved, and assesses their impact in the context of LWSNs.

Non-optimal tuning of these parameters at both the node and network-wide levels can lead to energy wastage in WSNs. Therefore, careful consideration and optimization of these parameters are essential to maximize energy efficiency and prolong the network’s lifetime. The main sources of energy wastage in WSNs (and LWSNs) include idle listening, overhearing, over-transmitting, packet collision, interference, control packet overhead, redundant data, etc. [57,58].

Now that we have analyzed the energy consumption of a sensor node and identified the parameters influencing WSN energy consumption at both the node and network-wide levels, our next step is to review the existing taxonomies for classifying the energy management techniques aimed at prolonging the WSN’s lifetime.

### 3.2. A Review of Taxonomies for Classifying Energy Management Techniques in WSN

A number of earlier studies have reviewed the energy management techniques for prolonging the WSN’s lifetime as follows: [34,36,37,38,56,57,59,60,61,62,63,64,65]. Most of these studies have developed a taxonomy for classifying the energy management techniques for prolonging the WSN’s lifetime. However, there is no generally accepted taxonomy for classifying energy management techniques prolonging the WSN’s lifetime. In this subsection, we discuss the existing studies that have reviewed the energy management techniques for prolonging the WSN’s lifetime and their taxonomies, and finally, we present an adapted taxonomy. Our adapted taxonomy will then permit us to classify the energy management techniques that have been used in existing studies to prolong the lifespan of LWSN monitoring systems.

Anastasi et al. [36] conducted a review on energy conservation in WSNs. They developed a taxonomy that broadly classified energy conservation schemes into techniques for minimizing energy consumption at the node level and techniques for minimizing energy consumption during network activities. The techniques for minimizing energy consumption during network activities include energy-efficient routing protocols and the implementation of mobile sinks, while the techniques for minimizing energy consumption at the node level include duty cycling techniques (radio optimization, sleep/wake-up schemes, transmission power control, dynamic voltage frequency scaling), and data-driven approaches (in-network processing, data compression, data aggregation, data prediction, hierarchical sensing, adaptive sampling, and model-based active sensing). All the techniques presented in this study are principally involved with energy savings, except for techniques such as mobile sink, energy-efficient routing protocols, and transmission power control, which can also be used for balancing the battery residual energy of the sensor nodes in the WSN. In another study, Rault et al. [38] carried out a review of energy efficiency in WSNs. They provided a taxonomy for energy management techniques that is similar to that presented in [36], except for the inclusion of battery repletion, which included energy-harvesting and wireless-charging techniques.

Singh et al. [65] developed a taxonomy that classified energy management techniques into battery management schemes, transmission power management schemes, system power management schemes, and miscellaneous. The battery management strategies include techniques that seek to leverage the internal characteristics of batteries to reclaim their charge, aiming to optimize the quantity of power supplied by the energy source. They range from node energy management schemes that dynamically vary the power supplied to the node depending on the workload to energy-balancing schemes that strive to attain a balance between the energy generated and energy consumed. The transmission power management schemes focus on techniques that restrict the transmission power of sensor nodes using parameters such as battery residual energy, energy-harvesting rate, etc. They range from the MAC layer management schemes to energy-aware routing schemes. The system power management schemes involve techniques that achieve a significant reduction in power consumption via efficient hardware design by using energy-efficient processors and peripherals that possess smart power-saving features. These techniques range from processor power management to device management. Finally, the miscellaneous schemes, according to the authors, involve techniques ranging from load balancing, duty cycling, mobile sink, and cross-layer optimization. The techniques presented in this study can be categorized into two main groups: techniques that seek to reduce the energy consumption at the node and network levels and techniques that seek to ensure a balance in the battery residual energy of all the sensor nodes in the network. The battery management and system management schemes seek to reduce the energy consumption at the node level and, therefore, belong to the former, while the transmission power management schemes and miscellaneous schemes, such as load balancing and mobile sink, seek to balance the residual battery energy of all the nodes in the network and therefore belong to the latter. This study did not discuss any techniques for prolonging the WSN’s lifetime by scavenging energy from external sources.

In [37], Engmann et al. reviewed techniques for prolonging the lifetime of the WSN. López-Ardao et al. [34] carried out a similar review, where they reviewed current trends in green wireless sensor networks. Both studies, [34,37], categorized energy management techniques for prolonging the WSN’s lifetime into energy conservation, energy harvesting, and energy transfer/charging techniques. The energy conservation techniques aim to extend the lifetime of the WSN by minimizing energy consumption at the sensor node level or network level while the WSN continues to operate as required. The energy-harvesting techniques aim to increase the energy available to the nodes by scavenging energy from the external environment such as solar, wind, vibrations, radio frequency, thermal, etc. The energy transfer/charging techniques aim to extend the lifetime of the WSN by engaging in wireless energy transfer from energy-rich nodes to energy-deficient nodes. Evangelakos et al. [57] broadly classified energy-saving methods into hardware-based and algorithmic-based energy-saving methods. The hardware-based methods encompass techniques like low-power sensors, low-power processors, low-power transceivers, energy harvesting, and wireless energy transfer. The algorithmic-based methods encompass techniques like data-driven approaches, duty cycling, and energy-efficient routing.

The studies of [62,66] classified energy management techniques for prolonging the WSN into two main groups: energy consumption and energy provision. According to the author, the former focuses on the operations and devices that deplete energy through performing transmission, reception, and data processing, whereas the latter intends to discover different methods for supplying the sensor node with the required energy source in order to allow the WSN to operate for as long as possible. From their taxonomy, it can be seen that the energy consumption schemes consist of energy-saving techniques (duty cycling and data-driven) and energy-balancing techniques (mobility-based), while the energy provision schemes involve energy-scavenging techniques (harvesting) and energy-balancing techniques (transference).

### 3.3. Adopted Taxonomy for Classifying Energy Management Techniques Is WSN

**Figure 5:**
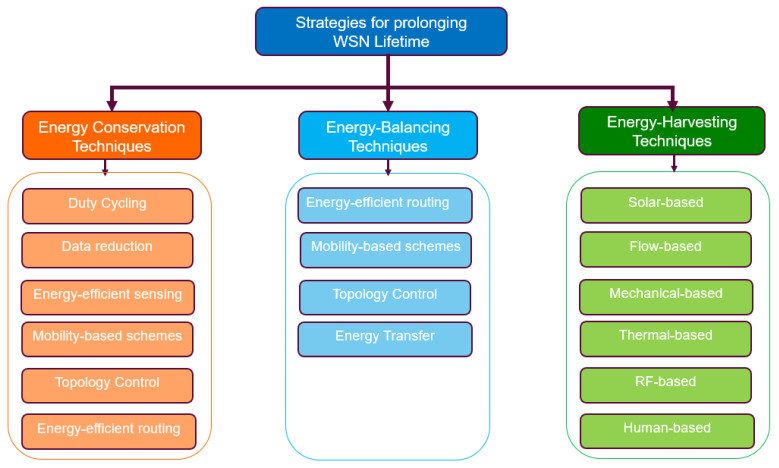
Taxonomy for classifying WSN lifetime prolongation strategies.

From surveying existing studies and their taxonomies for classifying energy management techniques for prolonging the WSN’s lifetime, we discovered that the lifetime of a WSN can be prolonged by either of the following three ways: reducing the energy consumed by individual sensor nodes or a group of sensor nodes, ensuring a balance in the battery residual energy of the sensor nodes in the WSN, or replacing the energy consumed in the batteries of sensor nodes. From this observation, we were able to develop a taxonomy that categorizes techniques for prolonging the WSN’s lifetime into three main categories: techniques that decrease the power consumption of individual nodes or the network (energy conservation techniques), techniques that provide a balanced energy consumption, i.e., balanced residual energy amongst sensor nodes (energy-balancing techniques), and techniques that replace the energy consumed in the batteries of sensor nodes (energy-harvesting techniques). Our adopted taxonomy is presented in Figure 5. The taxonomy presented in Figure 5 applies to traditional WSNs but can also be adapted to LWSNs. While many energy management techniques used in traditional WSNs will be similarly effective in LWSNs, those that rely on topology and routing may be less efficient or even ineffective due to the linear topology of LWSNs. We will analyze these specific cases in detail.

## 4. Energy Conservation Techniques

This section presents energy conservation techniques aimed at prolonging the lifespan of WSNs and evaluates their effectiveness in LWSNs.

**Figure 6:**
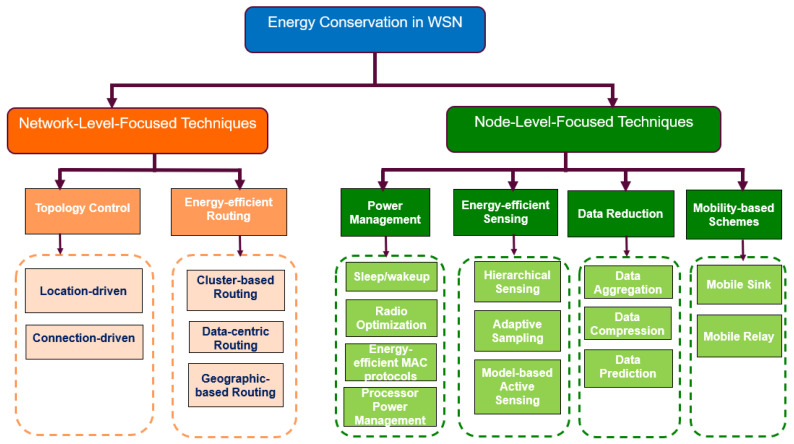
Taxonomy for classifying energy conservation techniques in WSNs.

Energy conservation techniques are principally involved with energy savings at the sensor node level or network level [59,60,64,67]. This can be done by implementing techniques that are aimed at minimizing energy consumption during network activities and/or implementing schemes that involve switching off node components that are not temporarily needed or dynamically adapting their power consumption [68]. The reason is that a large amount of energy is consumed by the node components (CPU, radio, sensor, etc.) even in idle mode [36]. Hence, energy conservation strategies primarily target minimizing the energy consumption of the communication, processing, and sensing subsystems to extend the lifetime of WSNs. Figure 6 presents a taxonomy we developed for classifying the energy conservation techniques that extend the WSN’s lifetime. In our proposed taxonomy, we broadly classified energy conservation schemes for extending the WSN’s lifetime into node-level-focused and network-level-focused techniques. On the one hand, node-level energy savings are achieved by reducing the energy consumption of the different sub-components of the sensor node (processor, transceiver, and sensors) via the use of duty cycling (power management), data-driven schemes (data reduction and energy-efficient sensing), or mobility-based schemes (mobile sink/relay). On the other hand, energy savings at the network level are achieved by reducing the energy consumption of a subset of nodes in the WSN via topology control schemes (location-driven and connection-driven schemes) or energy-efficient routing protocols (cluster-based, data-based, and geographic-based routing). The aim of the network-level energy-saving techniques is to maintain the network with a maximum node life [54].

Table 3 presents the different categories and sub-categories of energy conservation techniques for extending the WSN’s lifetime. For each technique, the strategy adopted, the components affected in order to achieve energy savings, the layer where the technique is implemented, and its suitability for LWSNs are presented. Table 4 presents an evaluation of the energy conservation technique discussed in this subsection. The pros and cons of each technique and an analysis of how the efficiency of these techniques could be applied to LWSNs are presented, while a detailed discussion is provided in Section 7. This can be helpful to engineers by assisting them in making the best decision during the design of energy-efficient LWSN monitoring systems.

**Table 3:** Rationale behind energy conservation techniques for extending WSN lifetime.

Energy Conservation Techniques		Strategy Implemented for Achieving Energy Savings	Targeted Components for Energy Reduction	Layer of Implementation	Suitability in LWSN
Power Management	Sleep/Wake-up	Reduces the energy consumption of the sensor node by applying duty cycling on the MCU, radio, and sensor, switching them between active and sleep modes.	MCU, radio, and/or sensor	Data Link/Application	Yes
	Radio Optimization	Reduces the energy consumption of the radio transceiver by dynamically adjusting radio parameters (coding and modulation rate, transmission power, antenna direction) using techniques such as transmission power control, cooperative communication schemes (SISO and MIMO), radio sleep/wake-up.	Radio	Physical	Yes
	Energy-Efficient MAC Protocol	Reduces the energy consumption of the radio by affecting radio idle listening, overhearing, over-transmitting, error control, retransmission rate, channel collisions, medium access, etc.	Radio	Data Link	Yes
	Processor Power Management	Reduces the energy consumption of the microprocessor by dynamically adjusting the power with respect to workload. It enables intelligent trade-offs between energy consumption and operational fidelity using techniques such as dynamic voltage scaling (DVS), dynamic voltage frequency scaling (DVFS), dynamic power management (DPM), etc.	MCU	Application	Yes
Data Reduction	Data Aggregation	Reduces the energy consumed during data transmission within the WSN by removing redundancies in the received data from the neighboring nodes and extracting the useful information by means of aggregation functions (maximum, minimum, average, etc.) as data travel over the network.	Radio	Application	Yes
	Data Compression	Reduces the energy consumed during data transmission by reducing the amount of data to be transmitted. This involves minimizing the number of bits required to represent each data block.	Radio	Application	Yes
	Data Prediction	Reduces energy consumption by minimizing the number of data transmissions. It involves predicting part of the sensed data without any transmission.	Radio	Application	Yes
Energy-Efficient Sensing	Hierarchical Sensing	Reduces energy consumption by trading-off accuracy for energy efficiency. It involves using low-power sensors to acquire coarse-grained information about the sensing field, with the power-hungry and more accurate sensors only activated when an event is detected.	Sensor	Application	Yes
	Adaptive Sampling	Reduces energy consumption by taking advantage of temporal and/or spatial correlations between sensed data.	Sensor and Radio	Application	Yes
	Model-Based Active Sensing	Reduces energy consumption by minimizing the number of data samples collected and transmitted.	Sensor and Radio	Application	Yes
Energy-Efficient Routing	Cluster-Based Routing	Reduces energy consumption by minimizing data transmissions thanks to data fusion, reduces communication range by limiting communication within the cluster, limits energy-intensive data fusion and coordination tasks to cluster heads, and selectively powers off other nodes in the cluster.	Network energy	Network	Yes
	Data-Centric Routing	Energy savings by eliminating data redundancy during transmission throughout the network.	Network energy	Network	Yes
	Geographic Routing	Reduces transmission energy by preferably using the shortest distance between nodes when maintaining or establishing a routing table.	Network energy	Network	No
Topology Control	Location-Driven	Reduces energy consumption by dynamically adapting the network topology based on the application needs and location of sensor nodes so as to allow network operations while minimizing the number of active nodes.	Network energy	Data Link	No
	Connection-Driven	Reduces energy consumption by dynamically deactivating some nodes while maintaining network operations and connectivity.	Network energy	Data Link	No
Mobility-Based	Mobile Sink	Energy savings thanks to reduced link errors, contention overhead, and forwarding range during communication.	Radio	Network/Application	Yes

**Table 4:** Evaluation of energy conservation techniques and their impact on LWSNs.

Energy-Saving Techniques	Key Parameters	Pros	Cons	Impact in LWSN
Low-Power Sensors [57]	Power efficiency, sensor accuracy.	Reduced energy consumption, extended battery life.	Limited sensing capabilities, potential compromise on data accuracy.	Similar effect as in traditional WSNs. More effective when combined with other techniques.
Duty Cycling [69,70]	Node synchronization, duty cycle scheduling.	Reduced energy consumption during periods of inactivity, extended battery life.	Potential impact on real-time data collection and responsiveness, increased latency in data transmission, and potential data loss.	Beneficial in LWSN as nodes in the linear topology can better synchronize sleep/wake cycles and reduce idle listening for efficient energy use.
Processor Power Management [55,68]	Processor workload predictability, task scheduling, and power control mechanisms.	Optimal energy consumption based on workload, improved battery life, enhanced performance efficiency.	Potential impact on processing performance or latency depending on power management settings, increased complexity in system design and management.	Less impactful in LWSN with predictable node behavior and little or no processing at the node level.
Radio Optimization [37,38,71]	Transmission range, radio power control, and interference management.	Reduced energy consumption during wireless communication.	Potential impact on communication range or reliability depending on optimization settings.	Transmission power control, sleep scheduling, and radio duty cycling are more straightforward and effective in LWSNs due to little variability in topology and radio parameters. Idle listening and overhearing are also reduced in LWSNs.
Energy-Efficient MAC Protocols [72]	Channel access control, collision management, idle listening, and retransmission interval.	Reduces unnecessary energy expenditure in communication.	Increased complexity in protocol design and overhead.	More effective in LWSNs due to the reduced complexity in communication coordination and the simplified linear topology. Less energy is wasted on idle listening, collision avoidance, and retransmissions.
Data Aggregation [73]	Data accuracy and processing load at aggregation points (DDNs, DRNs).	Reduces overall network energy consumption and minimizes data transmissions.	Potential loss of fine-grained data insights increases latency and requires more processing power at aggregation nodes.	Less effective in LWSNs due to fewer aggregation points along linear paths, resulting in modest energy savings.
Data Compression [74]	Compression ratio, computational load, and transmission power.	Lowers the energy cost of transmission by reducing the amount of data sent and decreasing bandwidth usage.	Compression may increase computational complexity, leading to higher processing power consumption.	More efficient in terms of energy consumption because of the simplified data flow and fewer nodes handling data aggregation tasks. The linear topology ensures that data are passed along a single path, reducing the need for re-compression or handling redundant data.
Data Prediction [75]	Prediction models and computational power.	Reduced data transmission cost.	Ineffective in highly dynamic environments with unpredictable data patterns, potential inaccuracies in prediction, complexity of model training	Effective in LWSN when monitoring environments where data trends are predictable, reducing transmission costs.
Edge Computing [21,76]	Local processing power and data transmission distances.	Reduces energy used for transmitting data over long distances.	Increases computational energy at the node level.	Beneficial in LWSNs as nodes can process data locally, minimizing long-distance transmissions via multi-hop communications in the linear path.
Hierarchical Sensing [77,78]	Sensor accuracy and power consumption.	Reduced energy consumption by frequently using low-power sensors monitoring when events of interest have not occurred.	Complexity in coordinating sensors with different capabilities.	Similar effect as in traditional WSN. More effective when combined with other techniques.
Adaptive Sampling [79]	Sampling rate, environmental monitoring sensitivity, and node synchronization.	Reduces unnecessary data collection and energy consumption by only sampling when necessary.	May miss critical events due to infrequent sampling; requires effective prediction or detection mechanisms.	Highly effective in LWSN for conserving energy when monitoring uniform conditions, but risks losing important data in event-driven applications.
Model-Based Active Sensing [80]	Model accuracy and event prediction capability.	Reduced sensor activation, minimized energy consumption.	Model accuracy is critical; incorrect predictions may lead to energy waste or missed events.	Highly effective in LWSNs for long-term monitoring scenarios with predictable environmental conditions.

### 4.1. Node-Level-Focused Energy Conservation Schemes

The node-level-focused energy conservation techniques can be categorized into duty cycling, data-driven, and mobility-based schemes. In this subsection, we describe the different energy-saving strategies that can be implemented using these techniques.

#### 4.1.1. Power Management Techniques

A sensor node exhibits various operational modes, which are determined by the states of its individual components, namely the processor, sensing subsystem, and radio transceiver. Each of these states is associated with distinct levels of power consumption. The term duty cycle is defined as the fraction of time a node is active during its lifetime. Thus, duty cycling techniques reduce the sensor node’s energy consumption by turning off the sensor node’s hardware components when they are not needed and waking them up whenever necessary [81]. This establishes a small duty cycle for the nodes based on the events occurring in the monitored environment [61]. Thus, techniques based on duty cycling rely on the fact that active nodes do not need to maintain their radios, processors, and sensing devices as being continuously on. According to the survey by Anastasi et al. [36], duty cycling is achieved by two complementary approaches, with one approach taking advantage of the redundancy in WSNs by adaptively selecting only a minimum subset of nodes to remain active for maintaining connectivity while the other approach ensures that the active nodes do not maintain their radio and sensors as being continuously on by constantly switching them off (i.e., placed in the low-power sleep mode) when there is no network activity. The authors termed the former topology control and the latter power management. Energy savings in WSNs via power management can be achieved through strategies such as adaptive sleep/wake-up, radio optimization, energy-efficient MAC protocols, and processor power management.

The strategy adopted by sleep/wake-up schemes is to reduce the energy consumption of the sensor node by applying duty cycling on the MCU, radio, and sensor, switching them between active and sleep modes. As generally known in the literature, the communication unit (radio module) consumes most of the sensor node’s energy [1,47]. By reducing the activities (transmission, reception, idle listening) of the radio module, higher energy savings can be achieved at the node level. Detailed information about this strategy is found in [36,57,59,61].

Several factors affect the power consumption characteristics of a radio module, including the radio duty cycle, modulation scheme, data rate, and transmission distance [59]. To optimize the radio and minimize sensor node energy consumption, researchers have developed radio optimization techniques that focus on optimizing radio parameters such as radio coding and modulation, transmission power, and antenna direction [37,38], using techniques such as adaptive transmission power control, dynamic frequency selection, duty cycling and low-power listening, smart antenna systems, etc. These techniques are implemented in energy-efficient cognitive radio [57]. Detailed information about these techniques is found in [36,38,82,83,84,85].

Energy-efficient MAC protocols impact various aspects, such as radio idle listening, overhearing, over-transmitting, error control, retransmission rate, channel collisions, and medium access [34]. Notably, their focus is often directed towards optimizing communication links between neighboring nodes rather than considering the broader network context. Consequently, mechanisms ensuring data reliability, including error detection and correction techniques, can be leveraged to achieve energy savings. The MAC layer protocols can be categorized into contention-less, contention-based, and hybrid protocols [36]. Singh et al. [65] presented a review of energy-efficient MAC layer protocols that have been used in WSNs.

Processor management techniques reduce the energy consumption of the microprocessor by dynamically adjusting the power with respect to the workload. They enable intelligent trade-offs between energy consumption and operational fidelity using techniques such as dynamic voltage scaling (DVS), dynamic voltage frequency scaling (DVFS), dynamic power management (DPM), hardware acceleration, etc. [55]. Detailed information about these techniques is found in [61,63,86]

Power management techniques, such as sleep/wake-up strategies and energy-efficient MAC protocols, are effective in LWSNs due to the minimal overhead associated with the node synchronization imposed by the linear topology. However, these techniques have a limited impact on reducing the number of multi-hop communications. In contrast, radio optimization techniques are more effective in LWSNs because the linear topology favors the use of directional antennas (beamforming in the 0° and 180° directions), which increase antenna gain without raising power consumption. This is particularly advantageous in LWSNs, especially when coverage needs to be extended due to the failure of a direct neighbor along the linear path. Traditional WSNs, on the other hand, typically require omnidirectional antennas, which consume more energy in long-range communications compared to directional antennas. Processor power management schemes are less effective in LWSN applications, where the processing load of the sensor nodes remains constant, and minimal processing is performed at the sensor node level.

#### 4.1.2. Data-Driven Techniques

The classification of data-driven techniques involves two main categories: data reduction and energy-efficient data acquisition schemes, each addressing specific challenges in WSNs [36,87,88]. Data reduction schemes primarily focus on minimizing the number of data transmissions and the volume of data transmitted as information travels from the sensor nodes to the base station. Conversely, energy-efficient data acquisition schemes concentrate on decreasing energy consumption within the sensing subsystem, often achieved by reducing the number of samples generated by the sensors.

Data reduction schemes in data-driven approaches are primarily executed through in-network processing within the WSN. In-network processing involves distributed computing, where data are processed as it traverses through the WSN towards the sink. This process includes tasks such as the fusion and aggregation of data as it progresses from one sensor node to another, effectively reducing the amount of redundant data that needs to be transmitted. Various techniques exist for in-network processing, with popular ones including data aggregation, data compression, and data prediction [34,36,37,65,88,89]. Data aggregation techniques play a crucial role in extending the network lifetime by efficiently merging data as it traverses through the network from one node to another until it reaches the sink [90,91]. Similarly, data compression involves encoding information at the sensor nodes and decoding it at the sink, effectively reducing the volume of data transmitted by source nodes [36]. This reduction in data transmission not only decreases the radio module’s active time but also reduces the energy consumption of sensor nodes. Additionally, data prediction techniques aim to minimize energy consumption by minimizing communication costs. These techniques create a model describing the evolution of sensed data, enabling the prediction of sensor node values within specified error bounds. The prediction model is deployed both at the sensor nodes and at the sink [57]. Transmissions between nodes and the sink occur only when the sensor node measurements deviate from the prediction model’s threshold [37], thereby reducing the transmission frequency and communication energy consumption.

The category of energy-efficient data acquisition data-driven schemes encompasses adaptive sensing techniques, including hierarchical sensing, adaptive sampling, and model-based active sensing [57,61]. These techniques aim to reduce the number of samples generated by sensors, thereby minimizing the amount of data to be processed and potentially transmitted by sensor nodes, leading to energy savings. In hierarchical sensing, a sensor node is equipped with multiple sensing devices that monitor the same physical parameter, each offering varying levels of sensing accuracy and power consumption. This setup allows for a trade-off between accuracy and energy efficiency, as lower-power sensors can provide a rough estimate of the monitored parameter [78]. Once an event is detected, higher-accuracy, power-hungry sensors can be activated to provide more precise readings, albeit at the expense of increased energy consumption. Adaptive sampling strategies are designed to minimize the number of measurements and communications required to achieve an accurate estimate by leveraging spatio-temporal correlations within the sensed data. These strategies involve dynamically adjusting the sampling rate and activating only a subset of sensors, thereby reducing the energy consumption associated with sensing [92]. Spatial correlation exploits the fact that measurements from sensor nodes in close proximity exhibit minimal differences. By activating only a few sensors in spatially correlated regions, energy expenditure on sensing can be reduced significantly. Temporal correlations, on the other hand, rely on the observation that if the monitored phenomenon changes slowly over time, the sampling rate can be decreased without sacrificing relevant information [36]. This allows for further energy savings by reducing unnecessary sampling activities during periods of stability [79]. Model-based active sampling aims to minimize the number of data samples by leveraging computational models. This approach involves the utilization of forecasting models to create an abstraction of the sensed phenomenon. Initially, the model is constructed using a set of sampled data, after which it is employed to predict future data points rather than continuously sampling in the field. By relying on these predictive models, the energy expended on data sensing and transmission can be conserved, leading to significant energy savings [80].

Data reduction schemes like data compression, data prediction, and edge computing are effective in LWSNs as they can greatly reduce the number of multi-hop communications. However, the gain of data aggregation in LWSNs is less effective compared to that of the traditional WSNs. This is because the linear topology prevents the creation of large clusters. Also, node redundancy in LWSNs is minimized by the linear topology. For energy-efficient data acquisition, hierarchical sensing and model-based active sampling have the same effect in LWSNs as in traditional WSNs, as they do not affect the number of multi-hop communications and are not affected by the linear structure of the network. The impact of adaptive sampling will be less effective in event-driven LWSN applications due to the linear topology, which does not benefit from spatio-temporal correlations.

#### 4.1.3. Mobility-Based Techniques

The mobility-based schemes use a few mobile nodes to achieve energy conservation in the network [62]. They leverage the mobility of nodes to dynamically adapt to changing environmental conditions and network requirements, thereby contributing to a prolonged WSN’s operational lifespan. By moving the sink or relay closer to the sensor nodes, they achieve energy savings thanks to reduced link errors, contention overhead, message loss, and forwarding range during communication. This eliminates multi-hop communications and reduces the energy consumed by the radio of sensor nodes. Mobility-based energy saving is achieved via a mobile sink and/or mobile relay [65]. Integrating mobile sinks or relays into WSNs introduces dynamic data collection mechanisms. Mobile sinks, equipped with enhanced processing capabilities, can intelligently navigate through the sensor field to collect data directly from nodes. By minimizing the need for extensive data transmissions and long communication routes, this mobility-based approach significantly reduces the energy expenditure of individual sensor nodes.

The impact of mobility-based schemes is highly effective in LWSNs compared to traditional WSNs because mobility-based schemes greatly reduce the number of multi-hop transmissions, which is the main source of energy wastage in LWSNs.

### 4.2. Network-Level-Focused Energy Conservation Schemes

Network-level-focused energy conservation schemes refer to strategies that are specifically designed to optimize energy consumption at the network level in the WSN. These schemes aim to enhance the overall energy efficiency and prolong the operational lifetime of the network by considering interactions and communication patterns among nodes. The rationale behind network-level-focused energy conservation schemes is grounded in minimizing energy wastage, improving communication reliability, and promoting sustainability. Network-level-focused energy conservation schemes are principally focused on reducing the energy consumption of the entire network rather than individual nodes. They may involve duty cycling schemes that can deactivate a subset of sensor nodes (topology control) or energy-efficient routing algorithms that reduce energy consumption by being energy-aware when routing data in the WSN.

#### 4.2.1. Topology Control

Topology control is a duty cycling scheme at the network level that takes advantage of network redundancy to prolong network longevity by dynamically adapting the network topology based on the application needs to minimize the number of active nodes [34]. It leads to turning off a subset of nodes while maintaining another subset of active nodes. This is because when sensors are redundantly distributed to provide good coverage, not all the nodes will be required to maintain network operation and connectivity [57]. It is thus possible to turn off the nodes that are not needed to ensure coverage or connectivity in order to prolong the lifetime of the WSN [93].

Topology control protocols can be generally classified into two main categories: location-driven and connectivity-driven approaches [36]. In the location-driven approaches, the activation or deactivation of nodes is determined by their specific locations. The assumption underlying these approaches is that the nodes’ positions are already known, enabling them to collaborate in deciding which nodes within a specific area should be deactivated without compromising the coverage of that particular area. Connectivity-driven protocols dynamically enable or disable sensor nodes to ensure the fulfillment of network connectivity or complete sensing coverage.

Topology control (location-driven and connection-driven) is not suitable in thin LWSNs because of the absence of node redundancy imposed by the linear structure. As such, all the nodes, not a subset of the nodes, are needed to maintain coverage.

#### 4.2.2. Energy-Efficient Routing

The goal of routing is to establish an effective path for data exchange between sensor nodes and the base station. This introduces certain overheads and entails energy consumption, particularly for the nodes in close proximity to the base station. In WSNs, a key design consideration for routing algorithms revolves around ensuring energy efficiency. Energy-efficient routing in WSNs refers to the implementation of routing protocols and strategies that optimize the energy consumption of sensor nodes during data transmission. This requires the design of routing mechanisms that minimize energy dissipation, reduce communication overhead, and ultimately extend the operational lifetime of the entire WSN.

Energy-efficient routing techniques play an important role in optimizing communication processes, minimizing energy consumption, and ultimately extending the lifetime of WSNs. In the literature, three notable strategies for achieving energy savings at the network level are cluster-based routing, data-centric routing, and geographic-based routing [38,62,65].

Cluster-based routing organizes the sensor nodes into clusters, with each cluster having a designated cluster head. Nodes within a cluster communicate with their respective cluster head, which, in turn, communicate with the base station or sink. This hierarchical structure facilitates energy savings through localized communication and efficient data aggregation. The rationale behind cluster-based routing lies in reducing the overall communication distance, minimizing the number of node-to-sink transmissions, and enabling energy-aware management within clusters. By aggregating data at the cluster level before forwarding it to the sink, redundant transmissions are avoided, leading to energy savings [94,95,96].

Data-centric routing revolves around the concept of organizing communication around the data rather than the nodes. Nodes collaborate to efficiently route data toward the sink based on the content or attributes of the information being transmitted. The rationale for data-centric routing is to minimize unnecessary transmissions by focusing on the data of interest. This strategy is particularly useful in applications where specific types of data need to be gathered or monitored, as it optimizes energy consumption by transmitting only relevant information. Data-centric routing schemes provide benefits such as selective data transmission and enhanced energy efficiency [97].

Geographic-based routing relies on the spatial information of nodes to determine the optimal path for data transmission. Nodes use location data to forward information towards the destination, often leveraging geographic coordinates or proximity-based strategies [98]. The rationale behind geographic-based routing is to exploit the physical positions of the nodes to minimize communication distances. This approach is particularly beneficial in scenarios where the geographic location of the nodes correlates with the efficiency of communication paths. Geographic-based routing schemes provide benefits such as shorter communication paths, adaption to node mobility, and scalability.

The impact of cluster-based routing is less effective in LWSNs compared to traditional WSNs because the number of multi-hop transmissions is not greatly reduced since the linear topology prevents the creation of large clusters. Data-centric routing can be effective in LWSNs as it has the capability to reduce the number of multi-hop communications. Geographic routing has little or no effect on LWSNs because of the limited routing options in LWSNs imposed by the linear topology. Routing in LWSNs is more concerned with reliable data delivery rather than the shortest path since there are just two routing options in LWSNs: forwarding to the left or forwarding to the right.

## 5. Energy-Balancing Techniques

This section presents the energy-balancing techniques aimed at prolonging the lifespan of WSNs and evaluates their effectiveness in LWSNs.

**Figure 7:**
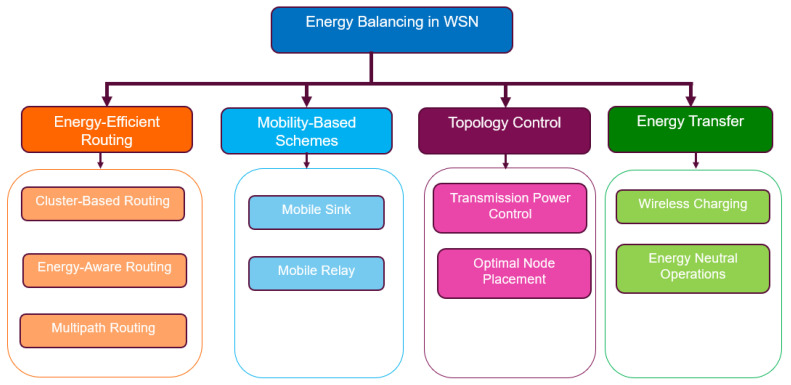
Taxonomy for classifying energy-balancing techniques in WSNs.

To achieve a longer network lifespan, both efficient and balanced power consumption are highly significant [65]. Energy-balancing techniques seek to ensure that the energy consumption is evenly distributed in the WSN so that the nodes have a fairly equal amount of energy. This reduces the likelihood of a black hole (energy hole) developing in the WSN and prolongs the WSN’s lifetime [37]. Thus, the objective of the energy-balancing technique is to balance the communication burdens of the sensor nodes in the WSN by ensuring that they spend their energy at approximately the same rate. The main rationale behind energy-balancing techniques is to maintain the same residual battery energy for all sensor nodes in the network. This can be achieved via energy-efficient routing schemes, load balancing, mobility-based schemes, topology control, and wireless energy transfer/charging. Figure 7 presents a taxonomy we developed for classifying the energy-balancing techniques for extending the WSN’s lifetime. Table 5 presents the different energy-balancing techniques and the strategies they employ. Table 6 presents an evaluation of the energy-balancing technique discussed in this subsection. The pros and cons of each technique and an analysis of the impact of these techniques when applied to LWSNs are presented.

### 5.1. Energy-Efficient Routing Protocols

Energy-efficient routing protocols are needed for large-scale battery-powered WSNs to ensure uniform energy consumption and load balancing. Moreover, they also need to achieve reliable and real-time data forwarding to the sink. This has led to many research efforts devoted to the design of energy-efficient routing protocols and/or enhancement of existing ones [63]. In the literature, two prominent strategies, cluster-based routing and energy-aware routing, are particularly effective in addressing the challenge of energy imbalance.

**Table 5:** Rationale behind energy-balancing techniques for extending WSN lifetime.

Energy-Balancing Technique		Strategy Implemented for Achieving Energy Balancing	Target	Suitability in LWSN
Energy-efficient routing	Load balancing (cluster-based routing)	Organizes the network into clusters, where each cluster is managed by a selected node known as the cluster head (CH) and balances energy consumption among sensor nodes via CH rotation. The selection is dynamic, and it is based on the residual energy. The node with the highest residual energy is selected as CH.	Network energy	No
	Energy-aware routing	Balances energy consumption among nodes by considering the residual energy when selecting the next hop during the setup path phase.	Network energy	No
	Multi-path routing	Balances energy among nodes by alternating forwarding nodes.	Network energy	No
Mobility-based approach	Mobile sink	Balances the load between nodes by using a mobile base station, which moves around the network to collect node information.	Network energy	Yes
	Mobile relay	Balances energy among nodes by introducing special mobile nodes to offer the service of message relaying.	Network energy	Yes
Topology control	Optimal node placement	Improves energy balance between nodes via optimal placement of nodes through even distribution or by adding a few relay nodes with enhanced capabilities.	Network energy	Yes
	Transmission power control	Balances energy consumption among nodes by allowing nodes to dynamically adjust their transmission power levels without losing connectivity. Nodes can increase or decrease their transmission power based on factors such as proximity to the destination and the necessity to reach neighboring nodes.	Network energy	Yes
Energy transfer	Wireless charging	Balances energy among nodes by wireless transmitting energy from energy-harvesting sources or nodes with high residual energy to nodes with low residual energy.	Network energy	Yes
	Energy-neutral operation	Balances energy consumption by achieving the desired network performance that can be supported by the energy harvested from the required energy sources (i.e., solar, vibration, and RF) and the network-wide operations (i.e., routing, clustering, and duty cycling) over longer periods of time.	Network energy	Yes

**Table 6:** Evaluation of energy-balancing techniques and their impact in LWSNs.

Energy-Balancing Techniques	Key Parameters	Pros	Cons	Impact in LWSN
Optimal node placement [99]	Node density, communication range, and message density.	Balances communication energy by optimizing the distance between nodes, thereby optimizing energy distribution.	Complex planning process; may not adapt well to dynamic environments.	Effective in LWSNs, but placement must account for the linearity of the network, message, and node densities to ensure uniform energy consumption.
Transmission power control [100]	Transmission power level and distance between nodes.	Reduces energy consumption by transmitting at the minimum required power level; lowers interference.	Requires real-time power adjustments; overhead in measuring node distances.	Very effective in LWSNs, as adjusting power for short-range communications between adjacent nodes minimizes energy waste. Also useful in achieving reliability during node failure by increasing the transmission power to jump over the failed sensor node.
Clustering [101]	Node-to-cluster head distance, cluster size, and number of clusters.	Reduces energy consumption through data aggregation; improves scalability.	Uneven energy depletion if cluster heads are not rotated properly; overhead in maintaining clusters.	Limited effectiveness in LWSNs due to fewer nodes in each cluster imposed by the linear topology; optimal cluster head placement is difficult, and cluster formation may be inefficient in long-distance deployments.
Energy-efficient routing protocols [102]	Residual energy and routing overhead.	Prolongs network lifetime by preventing overuse of specific nodes.	Suboptimal routing decisions may increase overall energy consumption; increased computational overhead.	Limited effectiveness in LWSNs due to fewer path options in LWSNs imposed by linear topology.
Multipath routing [103,104]	Path length, number of alternative paths, and route maintenance.	Increases fault tolerance and reliability; balances energy use across multiple nodes.	Increased overhead in maintaining multiple routes; higher complexity.	Less effective in LWSNs due to the minimal path options, resulting in suboptimal load balancing.
Mobile sink [105]	Sink mobility pattern and data collection frequency.	Reduces communication energy consumption by bringing the sink closer to nodes; prolongs network lifetime.	Increased complexity in sink movement coordination; may introduce delays.	Very effective in LWSNs by reducing the burden on edge nodes (greatly reduces the number of multi-hop communications).
Energy neutral operation [106,107]	Harvesting efficiency and energy availability.	Sustainable long-term operation without battery replacement or external power.	Harvested energy may be inconsistent; requires energy-harvesting hardware.	Viable for LWSNs, but effectiveness depends on the availability of energy-harvesting resources along the linear path.
Wireless energy transfer [108,109]	Energy transfer efficiency, charging frequency, energy replenishment rate, and positioning of charging stations.	Extends network lifespan indefinitely; reduces operational costs.	Requires specialized infrastructure, limited by charging range and efficiency, susceptible to interference.	Effective in LWSNs if charging stations are well-placed along the linear path, but challenges in coverage over long distances remain.

Cluster-based routing organizes sensor nodes into clusters, typically with a designated cluster head (CH). The CH performs specialized functions such as data fusion and aggregation and communicates the aggregated data directly to the base station or to other CHs. The CH can be selected randomly or based on one or more criteria, and this also largely affects the WSN’s lifetime. An ideal CH is the sensor node with the highest residual energy, the maximum number of neighbor nodes, and the smallest distance from the base station [110]. The goal of clustering schemes is to reduce the number of redundant communications in the WSN by reducing the number of nodes that communicate with the base station. By performing aggregation on data within the cluster, the energy consumed in the network is far less than when all the raw data are sent to the base station [111]. The rationale behind this approach is rooted in the desire to reduce overall communication distances, minimize energy consumption, and distribute energy-intensive tasks among the nodes effectively.

Energy-aware routing is another popular energy-balancing technique that has the ability to achieve uniform energy consumption. Energy-aware routing involves making routing decisions based on the current energy levels of sensor nodes. Nodes with higher residual energy are favored in routing decisions to evenly distribute the energy consumption across the network. The rationale is grounded in the necessity of preventing certain nodes from depleting their energy quickly, thus avoiding premature network failure.

As shown in Table 6, the energy-efficient routing techniques (cluster-based, multi-path, and energy-aware routing) primarily designed for traditional WSNs are less effective in LWSNs due to the lack of multiple routing paths imposed by the linear topology.

### 5.2. Mobility-Based Techniques

In a typical large-scale WSN, the base station (sink) is static. As such, the data from the sensor nodes are transmitted to the base station through multi-hop communications. Hence, some sensor nodes in the WSN would not only sense and send their data but also act as wireless relays that forward the data of their neighbors toward the sink. Consequently, nodes near the sink experience battery depletion faster, leading to nonuniform energy consumption, which eventually causes the development of an energy hole in the WSN. The energy hole disables the WSN and thus reduces its lifetime regardless of the fact that there are still a number of sensor nodes in the WSN whose batteries are not yet depleted.

In recent years, contrary to static sink, the mobile sink approach has attracted much research interest because of the increase in its potential WSN applications and its potential to improve network performance, such as energy efficiency and throughput [112]. Mobile sink schemes involve the deployment of a mobile sink or data collection point that moves through the network to gather data from sensor nodes. The rationale behind this approach is to redistribute the energy burden by moving the sink closer to the nodes with lower residual energy, allowing the sensor nodes to transmit data opportunistically when the sink is in proximity. This reduces the need for long-distance transmissions and provides relief to the nodes with higher energy consumption. Thus, the movement of the sink within the network helps to uniformly spread the energy consumption [36]. However, this solution is not very common since the sink in most WSN applications is static.

Mobile relay schemes involve the use of mobile nodes as relays to assist in data forwarding. These mobile relays move strategically to areas with higher energy consumption or congestion, providing temporary support and alleviating the burden on stationary sensor nodes. The rationale is to dynamically adjust the network topology, enabling efficient data relay and reducing the energy strain on specific nodes.

As shown in Table 6, mobility-based techniques are highly effective in LWSNs, as they significantly reduce the need for multi-hop communication, which is prevalent due to the constraints imposed by the linear topology.

### 5.3. Topology Control

Efficient topology control is instrumental in balancing energy consumption among the sensor nodes in WSNs, thereby extending the overall network lifetime. In this section, we discuss topology control techniques with a focus on balancing the residual energies of sensor nodes in the WSN. They are different from the topology control techniques presented in Section 4.1.2 (topology control techniques, which are focused on achieving energy savings at the network level) as their focus is to balance the residual energy of sensor nodes in the WSN [36].

The primary aim of this type of topology control technique is to improve the overall performance of the WSN by balancing energy consumption, reducing interference, enhancing network connectivity, and ultimately prolonging the lifetime of the sensor nodes. This type of topology control primarily makes use of radio optimization techniques at the node level to achieve its goal [100]. This involves controlling the transmission power and connectivity of individual sensor nodes to create an efficient and energy-aware network structure [113]. The rationale for this type of topology control includes energy efficiency, network connectivity, interference mitigation, and load balancing. By adjusting the transmission power levels, nodes can avoid unnecessary interference, collisions, and packet loss, leading to more reliable communication and reduced energy wastage.

Topology control can be implemented through strategies such as transmission power control and optimal node placement in order to achieve network-wide energy savings. Through transmission power control, nodes adjust their transmission power to limit the communication range based on the proximity of neighboring nodes and the communication requirements. This results in energy savings for the nodes with higher energy consumption, which in turn leads to energy balancing among sensor nodes and an extended WSN lifetime. Also, topology control may involve dynamically adjusting the positions of sensor nodes based on environmental changes or specific application requirements. Optimizing node placement can lead to more energy-efficient communication by adapting the node density based on the message density to achieve uniform energy dissipation [99].

As presented in Table 6, the linear topology of LWSNs makes transmission power control and optimal node placement particularly effective. Strategically placing nodes while considering message density and node distribution helps reduce the communication burden on critical nodes (those closest to the sink) and ensures uniform energy dissipation. Transmission power control is especially useful in LWSNs when a node along the linear path fails, as it allows the system to dynamically increase transmission power to bypass the failed sensor node.

### 5.4. Energy Transfer

Wireless energy transfer/charging schemes involve the transmission of energy from a source to a destination without the need for physical connections. In the context of WSNs, these schemes are designed to address energy imbalances among sensor nodes, thereby extending the overall WSN lifetime [114]. Wireless energy transfer techniques targeting energy balancing can be categorized into schemes that transfer via inductive coupling, magnetic resonant coupling, and electromagnetic radiations [37]. Inductive coupling relies on electromagnetic fields to transfer energy wirelessly between the coils embedded in sensor nodes [115]. When coils in close proximity resonate at the same frequency, energy is transferred from a source node to a destination node. Nodes with excess energy can transfer it to the nodes with lower energy levels, promoting a more balanced distribution of energy resources. Magnetic resonant coupling involves tuning coils to resonate at specific frequencies. This tuning enhances the range over which energy can be effectively transmitted. Magnetic resonant coupling allows for energy transfer over slightly longer distances compared to non-resonant methods [116]. Electromagnetic radiation, or EM radiation, emits energy from the transmitting antenna of a source to the receiving antenna through EM waves. This involves the transmission of energy through electromagnetic waves [117], typically in the form of RF or microwave signals. In the context of WSNs, these schemes harness electromagnetic radiation to wirelessly transfer energy between sensor nodes. The objective is to address energy imbalances, promote a more uniform distribution of energy resources, and extend the overall WSN’s lifetime.

Energy-neutral operations in the context of WSNs refer to the concept of achieving a balance between energy consumption and energy replenishment, ultimately maintaining a sustainable and continuous operation without depleting the energy reserves of sensor nodes [106]. The goal is to design WSNs in a way that the energy consumed during regular operations is offset by the energy harvested or received, resulting in a net-zero energy consumption over time [107]. Energy-neutral operations achieve energy balancing to extend the WSN’s lifetime by making use of strategies such as energy harvesting, dynamic power management, energy-aware communication protocols, and adaptive sensing strategies [118,119].

As presented in Table 6, energy transfer techniques can be as effective in LWSNs as they are in traditional WSNs.

## 6. Energy-Harvesting Techniques

This section presents the energy-harvesting techniques aimed at prolonging the lifespan of WSNs and evaluates their effectiveness in LWSNs.

While energy-saving techniques offer the potential to prolong the lifespan of WSNs, it is crucial to acknowledge that sensor nodes relying on batteries cannot ensure uninterrupted monitoring over extended durations. This constraint stems from the finite capacity of batteries, which inevitably deplete over time. This challenge is especially pronounced in WSN applications where nodes may be situated in physically inaccessible locations. In such cases, the logistical and cost-related obstacles associated with battery replacement become substantial. Despite the available techniques to mitigate power consumption, the reliance on battery power imposes constraints on the operational lifespan of the system, necessitating periodic battery replacements or recharging [120]. Additionally, the monitoring process is interrupted during battery replacement, resulting in downtime [121]. To address these limitations, energy-harvesting techniques emerge as a viable solution [48]. By harnessing ambient and/or external energy sources from the environment, these techniques offer a means to replenish the energy consumed by sensor node batteries [122]. Implementation of energy harvesting not only mitigates the constraints posed by a finite battery life but also enhances the sustainability and longevity of WSNs [49], especially in remote or difficult-to-access deployment scenarios like border surveillance and highway, railway, powerline, and pipeline monitoring.

Energy-harvesting (EH) techniques convert energy from external sources, which are non-renewable, or from ambient environment sources, which are renewable, into electrical energy that can be used to power autonomous devices such as wireless sensor nodes [37,122]. While numerous existing EH systems produce only a restricted amount of power, in the order of µWcm^−2^ to mWcm^−2^, the increasing popularity of EH is attributed to advancements in very low-power sensors and wireless communication systems [123]. The energy harvested from external and ambient sources is used to replenish the energy depleted by the sensor node. This ensures that the battery energy of the sensor nodes is not depleted and thus prevents the development of an energy hole in the WSN. This increases the lifetime of the nodes and that of the WSN as a whole, thus preventing frequent battery replacement in most applications. Thus, the goal of EH techniques is to convert energy from one form to another that can be used to power sensor nodes and thus extend the lifetime of the WSN [124].

**Figure 8:**
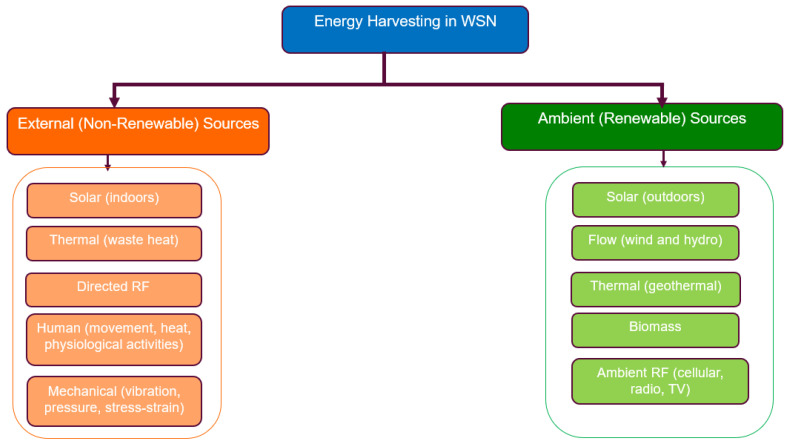
Taxonomy for classifying energy-harvesting techniques in WSN.

The source from which energy is harvested in a WSN is a valuable resource since it determines the amount of energy available to the network and the rate of conversion from the source to electrical energy [37]. This makes the ambient sources which are accessible within an environment and which do not need any external energy supply very attractive to WSN applications. Table 7 presents a comparison of the different energy-harvesting techniques based on energy sources, conversion materials, conversion mechanisms, typical power densities, and conversion efficiency. A taxonomy for classifying energy harvesters is presented in Figure 8. The ambient (renewable) sources consist of flow (wind and hydro), solar (outdoor), thermal (geothermal), and ambient RFs. These sources are also referred to as primary or renewable sources because they can be replenished over time through natural processes [48]. Energy harvesting from ambient sources plays a crucial role in prolonging the lifespan of WSNs for two primary reasons. Firstly, energy harvested from the environment is pollution-free, contributing to environmentally sustainable operations. Secondly, as a renewable resource, it offers the potential for devices to operate unattended for virtually unlimited periods, enhancing the autonomy and longevity of WSNs. The external (non-renewable) sources consist of solar (indoors), directed RF, thermal (waste heat), magnetic field, human (motion and temperature), and mechanical (vibrations, stress, and pressure) sources.

A detailed review of energy-harvesting techniques for the WSN and IoT can be found in [4,48,49,120,122,125,126]. The works of Singh et al. [122] and Williams et al. [49] presented a comprehensive taxonomic survey on recent energy-harvesting techniques in WSNs and a concise summary and comparative analysis of various promising techniques for energy harvesting. Sanislav et al. [4] and Elahi et al. [125] presented a review of recent advances in energy-harvesting techniques for IoT. From the literature, the most popular energy-harvesting techniques used in the WSN and IoT include solar-based, thermal-based, wind-based, vibration-based, and RF-based sources [37,124,127]. Prauzek et al. [124] reviewed and presented a comprehensive account of energy-harvesting sources, energy storage devices, and corresponding topologies of energy-harvesting systems, published from 2008 to 2018. In another study, Peruzzi and Pozzebon [127], in their review paper, provided a detailed overview of the existing low-power wide-area network (LPWAN) systems relying on energy harvesting for their powering. In [127], the different LPWAN technologies and protocols are discussed alongside the applicable energy-harvesting techniques and presentations of the architecture of the power management units.

**Table 7:** Classification of energy harvesters based on energy source, conversion materials, and conversion mechanism.

Energy Source	Transducer	Conversion Mechanism	Typical Power Density	Conversion Efficiency
Solar (Outdoor)	Solar panels	Photovoltaic effect	15 mW/cm^2^ [128]	15–25%
Flow (Wind)	Wind turbine	Electromagnetic induction	7.6 mW/cm^2^ @5 m/s [129]	30–50%
Flow (Hydro)	Turbine	Electromagnetic induction	N.A.	70–90% [130]
Thermal	Thermoelectric generator	Seebeck effect	15 µW/cm^3^ [125]	5–17%
Mechanical	Magnetostrictive materials	Magnetostriction	145 µW/cm^3^ [131]	10–50%
	Piezoelectric materials	Piezoelectric effect	4.57 mW/cm^3^ [132]	10–50%
	Electrostatic materials	Capacitance modulation	50 µW/cm^3^ [128]	10–50%
Directed RF	Antenna	Electromagnetic induction	50 mW/cm^2^ [131]	5–30%
Ambient RF	Antenna	Electromagnetic induction	2 µW/cm^2^ [133]	5–30%
Electric Field	Capacitive transducers	Electrostatic induction	0.04 µW/cm^3^ [134]	5–30%
Magnetic Field	Current transformers	Electromagnetic induction	100 mW/cm^3^ [135]	5–30%
Biomass	Microbial fuel cell	Bioelectrochemical conversion	300 µW/cm^2^ [136]	<1% [137,138]

Although EH techniques provide a viable solution to extending the lifespan of WSNs, they face some challenges, such as the stochastic nature of the energy sources [139,140,141]. If the source of the EH technique is abundant, then a sensor node can be powered continuously [48], thereby enabling perpetual operation. However, since most energy sources are discontinuous and provide varied levels of energy at different times, sensor nodes powered by EH must be designed to store the scavenged energy when the natural source is present for later use [120]. Table 8 presents the strengths and drawbacks of the different EH techniques presented in this subsection. It is important to note that these EH techniques are expected to have a similar impact in LWSNs as in traditional WSNs, as they are not affected by the linear topology of LWSNs.

## 7. Discussion

This section discusses the reasons why certain energy-saving techniques that work in traditional WSNs are not suitable for LWSN monitoring applications. It also presents a comparative analysis of the energy consumption in LWSNs and traditional WSNs and the integration of user requirements and operational constraints in LWSN energy management.

### 7.1. Challenges Adapting Energy Management Techniques from Traditional WSN to LWSN Monitoring Applications

To discuss why these techniques designed for traditional WSNs may not work for LWSNs, it is important to recap the difference between traditional WSNs and LWSNs. The main difference between WSNs and LWSNs lies in the deployment of the nodes. In typical WSNs, nodes are randomly deployed, whereas, in LWSNs, the network topology is predetermined. LWSNs necessitate a linear arrangement of sensor nodes, with data relayed to a central base station through daisy-chaining via neighboring sensor nodes. However, this approach introduces significant energy inefficiencies and imbalances, resulting in data transmission delays and complex application and service management [153]. While some energy-saving techniques have proven effective in traditional WSNs, their applicability in LWSN monitoring applications poses unique challenges [8]. Several factors contribute to the incompatibility of certain energy-saving techniques of traditional WSNs in the context of LWSNs [154]. In this section, we explore the reasons why certain energy-saving techniques that work in traditional WSNs are not suitable for LWSN monitoring applications.

#### 7.1.1. Node Distribution and Communication Range

In traditional WSNs, sensor nodes are typically deployed in a random or clustered fashion (most likely giving them a mesh-like topology), facilitating short-range communication and proximity-based data aggregation. However, in thin LWSN monitoring applications, sensor nodes are spread out along a linear path, leading to longer communication distances and increased energy consumption for data transmission [155]. Techniques relying on close proximity for efficient communication, such as node clustering, become less effective in thin LWSNs due to the dispersed node distribution and extended communication range. This makes it challenging for existing energy-balancing schemes (e.g., cluster-based, multi-path, and energy-aware routing) designed for traditional WSNs to be directly applicable to LWSNs due to the unique characteristics of the nodes in LWSNs [154]. To achieve reliable data delivery in large-scale monitoring applications, traditional WSNs make use of multi-hop routing. However, to effectively utilize the multi-hop approach in LWSNs, it is advisable to reduce the distance of the network by deploying multiple sink nodes [156,157,158]. This approach helps to mitigate the energy hole problem [159,160] and can also decrease the number of hops required for data transmission, thereby reducing the overall energy consumption. Also, a pure multi-hop approach to route data along the LWSN extending for hundreds or even thousands of kilometers can incur significant energy costs. As a result, data collection strategies employing mobile sinks [161], such as unmanned aerial vehicles (UAVs), are often more suitable compared to traditional WSN multi-hop routing approaches [41].

Another major challenge in LWSNs is to ensure end-to-end packet delivery with a smaller number of relay nodes [27,162]. This is because, unlike in traditional WSNs, nodes near the sink in LWSNs tend to deplete their energy more rapidly because they handle heavier traffic compared to other nodes in the network [21]. Over time, this uneven load distribution, commonly referred to as the “relay burden problem”, leads to a disproportionate consumption of energy, leaving the nodes in close proximity to the sink node with significantly less energy [163]. Consequently, the risk of prematurely depleting the network’s overall energy reserves and shortening its lifetime is greatly amplified. Moreover, these close-in nodes cannot afford extended sleep periods as they need to remain vigilant in idle listening mode to fulfill their relaying duties. Therefore, it becomes imperative to implement more intelligent methods like optimal node placement [99] and mobile sink [41] for distributing the traffic load across the network to ensure and prolong its operational lifetime.

#### 7.1.2. Scalability and Reliability

LWSN monitoring applications often span large geographical areas, necessitating scalable and reliable communication protocols for robust data transmission. However, techniques optimized for small-scale WSN deployments may encounter scalability and reliability challenges when applied to LWSNs [164]. For instance, geographic routing protocols, which rely on neighbor information and use the shortest distance between nodes for routing decisions, may struggle to maintain network connectivity and packet delivery ratios in linear deployments with sparse node distribution [165]. Additionally, the routing complexity in traditional WSNs contrasts with the straightforward routing paths dictated by the linear structure of LWSNs [40], where nodes have only two possible routing paths (left or right) [28]. Furthermore, techniques like topology control (location-driven and connection-driven) may not be suitable for LWSNs due to a lower spatial correlation in the data acquired by the nodes. Unlike traditional WSNs with random node deployment, thin LWSNs deploy nodes deterministically, resulting in fewer node redundancies [8]. As such, all the nodes, not a subset of the nodes, are needed to maintain coverage and operation.

While certain techniques effective in traditional WSNs may not be directly applicable to LWSNs, cross-layer approaches, which consider the energy consumption issue on a system-wide level rather than focusing solely on individual components or protocols, may be advantageous [166]. Cross-layer solutions can be utilized to effectively reduce energy expenditure across the entire protocol stack since the parameters and protocols across various layers of the protocol stack in WSNs are jointly optimized [38,167]. They achieve energy savings by making use of the information residing at different layers of the network protocol stack [168]. However, achieving cross-layer optimization, where parameters and protocols across different layers of the protocol stack are jointly optimized, is particularly challenging in LWSN monitoring applications. The linear topology and long-distance communication requirements introduce complexities that traditional cross-layer optimization techniques may struggle to address [162]. Therefore, innovative cross-layer approaches that account for the specific challenges and operational requirements of linear deployments can pave the way for efficient and sustainable energy management in LWSN monitoring applications.

#### 7.1.3. Energy Consumption and Efficiency

Energy-saving techniques in traditional WSNs are tailored to minimize energy consumption at both the node and network levels, optimizing resource utilization and extending the battery life. However, in LWSN monitoring applications, energy consumption is inherently higher due to the demands of long-distance communication and data forwarding. This is because, in a typical LWSN, a limited number of sensors are often deployed to collect data and forward it to the sink node. For such sensor nodes, energy is expended not only for long-range communications but also for multi-hop communication, where the nodes not only transmit their own data but also serve as relays for transmitting data from neighboring nodes [169]. Consequently, these sensors may experience higher energy depletion, impacting the overall network’s performance.

Techniques in traditional WSNs, which solely focus on node-level energy reduction, may overlook the energy-intensive nature of long-range communication in LWSNs. These techniques may not work well in linear deployments (which require tailored approaches to effectively balance energy consumption and network performance), resulting in suboptimal performance and a shortened network lifespan. Therefore, energy-balancing techniques that focus on overall network energy consumption and strive to balance the residual energy of sensor nodes are crucial in LWSN monitoring applications for extending the network’s lifetime [170]. In addition, optimal node placement is also crucial for achieving energy efficiency in LWSNs [162,171]. By strategically positioning sensor nodes along the linear deployment path, factors such as the communication range, node density, and energy consumption can be optimized to minimize energy expenditure and enhance the network’s performance [172].

#### 7.1.4. Environmental Constraints and Fault Tolerance

Linear WSN monitoring applications often operate in harsh and environmentally challenging conditions, where factors such as terrain, weather, and interference can impact a network’s performance and energy efficiency. Energy-saving techniques that rely on idealized assumptions about network topology and environmental conditions may fail to account for the real-world constraints encountered in LWSNs [30].

The linear topology inherent in LWSNs makes them more vulnerable to data delivery failures compared to traditional WSNs [27]. In LWSNs, a single node failure has the potential to disrupt the entire communication process, representing a significant weakness. Node failures can occur due to various factors, such as battery depletion, hardware malfunctions, or external damage. Additionally, the occurrence of consecutive faulty nodes can create gaps in the network, leading to the fragmentation of the LWSN into multiple disjointed segments [28]. Moreover, the failure of nodes located closer to the sink node, especially if they are overloaded, can expedite the depletion of the network’s energy resources, thereby shortening its operational lifespan. Such failures can have severe repercussions, emphasizing the critical importance of robust MAC and network layer protocols in LWSNs [26]. These protocols must be equipped to handle node failures effectively, as there are limited alternative routing options available, particularly towards the sink node.

### 7.2. Comparative Analysis of Energy Consumption in LWSNs and Traditional WSNs

Energy consumption in LWSNs follows unique patterns due to the linear topology, which often involves fewer transmission paths and restricted routing options compared to traditional WSNs. In traditional WSNs, sensor nodes are often arranged in a multi-dimensional array, allowing for multiple routing paths, which can distribute the energy load more evenly among nodes. This also permits them to efficiently utilize cluster-based aggregation to reduce redundant data transmission and conserve energy. However, in LWSNs, nodes are deployed linearly, with each node having only two immediate neighbors along the linear path, leading to specific energy demands and challenges, especially near the sink node, where the energy depletion rates are typically higher. Furthermore, data aggregation is less effective due to fewer opportunities for clustering along the linear path.

This study identifies that LWSNs consume more energy for multi-hop communication along linear paths, as data are relayed through the intermediate nodes with limited alternative routes. To mitigate these challenges, energy-efficient routing protocols specifically designed for linear topologies are recommended. These protocols should emphasize balancing energy consumption among nodes and managing transmission power to extend the lifespan of the nodes closest to the sink. Insights drawn from this comparison indicate that adopting directional antenna configurations, using mobile sinks (e.g., UAVs for data collection), and optimizing relay node placement can improve energy efficiency and reduce communication overheads, ensuring a more stable operational lifespan for LWSNs.

### 7.3. Integration of User Requirements and Operational Constraints in LWSN Energy Management

In designing energy management strategies for LWSNs, it is essential to align with the user’s requirements and operational constraints, such as network lifetime expectations, coverage demands, and cost limitations. Real-world applications of LWSNs, such as infrastructure monitoring and border surveillance, often require continuous, uninterrupted operation in remote and sometimes inaccessible locations. This necessitates strategies that are both energy-efficient and sustainable under practical conditions.

Integrating user requirements involves defining specific performance metrics, including desired monitoring frequency, data accuracy, and acceptable latency. Operational constraints, such as environmental factors (temperature and terrain) and maintenance accessibility, should influence the selection of energy-saving techniques. For instance, the adoption of adaptive strategies, such as duty cycling, hierarchical sensing, dynamic power adjustment, etc., can enhance energy efficiency without compromising data quality or network coverage. Furthermore, adopting energy-harvesting methods, where feasible, can ensure a consistent energy supply while employing dynamic power adjustment mechanisms to help meet the diverse energy demands posed by varying environmental conditions. These tailored energy management strategies will ensure that LWSNs remain functional and reliable, meeting the efficiency and practicality required for real-world deployments.

## 8. Conclusions

This paper explored various energy management strategies aimed at extending the lifespan of linear wireless sensor networks (LWSNs). By examining the challenges posed by linear deployments of WSNs, such as those encountered in border surveillance and road, bridge, railway, and pipeline monitoring, this study emphasizes the critical need for effective energy management solutions. It broadly classified energy management strategies for extending the lifespan of WSNs into three categories: energy conservation, energy-balancing technique, and energy-harvesting technique. Furthermore, it evaluates the suitability and impact of these techniques when applied to LWSNs, highlighting that not all approaches suitable for traditional WSNs are equally effective for linear configurations. Techniques such as clustering, topology control, energy-aware routing, geographic routing, etc., which rely on proximity-based communication and node redundancy, are found to be less suitable for LWSNs due to the linear arrangement of the nodes, large distances, and limited routing options. The adoption of effective green strategies is thus crucial for LWSN applications where energy efficiency and sustainability are paramount. By utilizing energy-saving and energy-harvesting techniques, LWSN monitoring systems can minimize operational costs, reduce the environmental impact, and extend a network’s lifetime.

## Figures and Tables

**Table 1:** Parameters affecting energy consumption in a sensor node.

Component Affected	Parameter
Sensing unit	Sensor duty cycle, sampling frequency, sensor resolution, sensor radius, sensor type
Processing unit (MCU, algorithms, protocols, OS, and applications, memory unit)	Supply voltage, operational frequency, MCU duty cycle, MCU operating modes, number of processed bits
	Channel time slot allocation, MAC duty cycle, message size, channel sampling interval
	Application parameters
	OS scheduling
	Number of stored bits, number of memory reads and writes, duration of storage
Communication unit	Transceiver duty cycle, transceiver operational modes, transmission power (transmission distance), modulation scheme, data rate, packet size, packet transmission interval, antenna direction

**Table 2:** Parameters influencing energy consumption at the network-wide level and their associated layers.

Layer Affected	Parameters	Influence in LWSN Configuration
Physical layer	Transmission power, transmission range, packet size, data rate, modulation scheme	The influence of these parameters on energy consumption is in much the same way as in traditional WSNs
Data link layer	Topology, duty cycle, synchronization, MAC protocol, error control mechanism, frame size, acknowledgment mechanism, neighbor discovery	The influence of these parameters on energy consumption may differ slightly from traditional WSNs due to the fixed linear topology of LWSNs since each node typically has two neighbors (one preceding and one following), making the topology predictable. Despite this, since the data link layer primarily handles point-to-point communication, its overall influence on energy consumption remains similar to that in traditional WSNs.
Network layer	Routing protocol, route discovery and maintenance, routing metric, multicast and broadcast operations, quality of service (QoS) requirements, maximum jump factor (MJF), fault detection and recovery	The influence of these parameters on energy consumption significantly differs from traditional WSNs. The fixed routing structure in LWSNs eliminates the flexibility found in traditional WSNs, where nodes can dynamically choose among multiple neighbors. Furthermore, broadcast operations, which are commonly used for route discovery and network maintenance in traditional WSNs, are largely absent in LWSNs due to the linear structure.
Transport layer	Retransmission rate, timeout interval, retransmission limit, window size, protocol overhead, security mechanism, traffic pattern	These parameters remain relevant in LWSNs, similar to traditional WSNs. However, the one-dimensional nature of LWSNs can limit the effectiveness of certain optimization techniques.
Application layer	Message size, task scheduling, data fusion and aggregation, data compression, data filtering, application protocol, data collection frequency, quality of service (QoS) requirements	Functions similar to those in traditional WSNs. Data fusion and aggregation strategies are still relevant and effective, but they are tailored to the specific roles of the different node types within the linear structure.

**Table 8:** Evaluation of energy-harvesting techniques for extending WSN lifetime.

Energy-Harvesting Technique	Description	Pros	Cons
Solar Energy Harvesting (outdoor) [142]	Harvests energy from sunlight using photovoltaic cells or solar panels.	Abundant energy source, ambient, environmentally friendly, relatively low maintenance, high output voltage, predictable, simple form factor, no moving parts, highly scalable, mature technology.	Dependent on sunlight availability and intensity, discontinuous, uncontrollable, significantly affected by weather conditions, dust, sand, dirt, ice, or snow, and high initial installation costs.
Thermal Energy Harvesting [143]	Converts temperature differences into electrical energy using thermoelectric materials or thermoelectric generators.	Utilizes waste heat and can operate in various environments with temperature gradients; continuous in appropriate environments.	Limited efficiency, requires significant temperature differences for effective energy generation, non-ambient, uncontrollable, unpredictable, variable output power.
Radio Frequency (RF) Energy Harvesting [144]	Captures ambient RF signals from sources like Wi-Fi, cellular networks, or RFID systems and converts them into electrical energy.	Ubiquitous RF sources, potential for continuous energy generation, scalable, predictable, no moving parts.	Limited power output, highly dependent on RF signal strength and availability, distance-dependent, uncontrollable.
Wind Energy Harvesting [145]	Harnesses wind energy using small turbines or wind turbines to generate electricity.	Clean and renewable energy source, potentially high-power output in windy areas, ambient, mature technology.	Site-dependent, discontinuous, difficult to apply form factor, hostile application environments, uncontrollable, unpredictable, limited in urban environments, noise pollution, and high initial installation and maintenance costs.
Hydro Energy Harvesting [146]	Utilizes the flow of water, such as rivers, streams, or tidal currents, to drive turbines or generators and convert kinetic energy into electrical energy.	Abundant renewable energy source, consistent and predictable energy generation, relatively high-power output, suitable for both large-scale and small-scale applications, minimal environmental impact, ambient.	Site-specific, requires access to flowing water sources, infrastructure-intensive, and high initial investment and maintenance costs, potential ecological disruption to aquatic habitats and ecosystems.
Vibrational Energy Harvesting (piezoelectric) [147]	Harvests energy from mechanical motion, such as vibrations or movements, using piezoelectric, electrostatic materials, or electromagnetic induction.	Harvests energy from various mechanical sources, potential for continuous energy generation, controllable, passive, simple.	Relatively low power output, requires significant vibrations or movements for effective energy harvesting, variable output, unpredictable, resonant frequency matching requirement, unresponsive at low frequencies, use of delicate materials, non-ambient.
Vibrational Energy Harvesting (electrostatic) [148,149]		High-power density, low cost, high output voltage, controllable.	Need for an external voltage source during operation (bias voltage required), unpredictable, non-ambient.
Vibrational Energy Harvesting (electromagnetic) [150]		High output current, low cost, robust, controllable, operates at low frequency.	Large size, unpredictable, low voltage, resonant frequency matching requirement, involves moving parts, non-ambient.
Electric Field Harvesting [123]	Utilizes electric field variations in the environment to generate electrical energy through capacitive coupling or electrostatic induction mechanisms.	No moving parts, suitable for indoor environments, potential for continuous energy generation, induced voltage can almost remain unchanged under normal operating conditions, ambient.	Relatively low power output, highly dependent on electric field strength and proximity to electric sources.
Magnetic Field Harvesting [151]	Utilizes variations in magnetic fields in the environment to induce electrical currents in coils or magnetic materials to generate electrical energy.	No moving parts, suitable for both indoor and outdoor environments, potential for continuous energy generation.	Relatively low power output, highly dependent on magnetic field strength and proximity to magnetic sources, limited range of applications.
Biomass Energy Harvesting (microbial fuel cells) [152]	Utilizes microbial processes to convert organic matter into electrical energy through biochemical reactions within microbial fuel cells (MFCs).	Utilizes organic waste as a renewable energy source, suitable for decentralized applications, potential for continuous energy generation.	Relatively low power output, influenced by environmental factors and organic matter availability, requires careful management of microbial communities and operating conditions, longer startup time.
